# Limited Migration From Physiological Refugia Constrains the Rescue of Native Gastropods Facing an Invasive Predator

**DOI:** 10.1111/eva.70004

**Published:** 2024-10-21

**Authors:** Mathilde Salamon, Louis Astorg, Antoine Paccard, Frederic Chain, Andrew P. Hendry, Alison M. Derry, Rowan D. H. Barrett

**Affiliations:** ^1^ Université du Québec à Montréal Montreal Quebec Canada; ^2^ McGill Genome Centre Montreal Quebec Canada; ^3^ University of Massachusetts Lowell Lowell Massachusetts USA; ^4^ McGill University Montreal Quebec Canada

**Keywords:** adaptation, aquatic invasive species (AIS), gastropods, genetic rescue, whole‐genome sequencing

## Abstract

Biological invasions have caused the loss of freshwater biodiversity worldwide. The interplay between adaptive responses and demographic characteristics of populations impacted by invasions is expected to be important for their resilience, but the interaction between these factors is poorly understood. The freshwater gastropod *Amnicola limosus* is native to the Upper St. Lawrence River and distributed along a water calcium concentration gradient within which high‐calcium habitats are impacted by an invasive predator fish (*Neogobius melanostomus*, round goby), whereas low‐calcium habitats provide refuges for the gastropods from the invasive predator. Our objectives were to (1) test for adaptation of *A. limosus* to the invasive predator and the low‐calcium habitats, and (2) investigate if migrant gastropods could move from refuge populations to declining invaded populations (i.e., demographic rescue), which could also help maintain genetic diversity through gene flow (i.e., genetic rescue). We conducted a laboratory reciprocal transplant of wild F_0_ *A. limosus* sourced from the two habitat types (high calcium/invaded and low calcium/refuge) to measure adult survival and fecundity in home and transplant treatments of water calcium concentration (low/high) and round goby cue (present/absent). We then applied pooled whole‐genome sequencing of 12 gastropod populations from across the calcium/invasion gradient. We identified patterns of life‐history traits and genetic differentiation across the habitats that are consistent with local adaptation to low‐calcium concentrations in refuge populations and to round goby predation in invaded populations. We also detected restricted gene flow from the low‐calcium refugia towards high‐calcium invaded populations, implying that the potential for demographic and genetic rescue is limited by natural dispersal. Our study highlights the importance of considering the potentially conflicting effects of local adaptation and gene flow for the resilience of populations coping with invasive predators.

## 1 Introduction

Invasive species represent a significant threat to global biodiversity (Early et al. 2016; Dueñas et al. 2021). They are an important driver of species extinction (Bellard et al. 2017) and can have a strong negative impact on native species abundance (Emery‐Butcher, Beatty, and Robson 2020). The strength of the impact of invasive species on native populations and communities depends on their abundance and trophic level, with invasive predators typically having the most substantial effects (Doherty et al. 2016; Bradley et al. 2019). Indeed, among natural and anthropogenic stressors, invasive predators are one of the strongest agents of selection driving phenotypic change in native populations (Sanderson et al. 2022). Environmental gradients can be important barriers that restrict invasive species' advance (Crespo et al. 2015; Mothes et al. 2019; Liu et al. 2020), their abundance, and thereby potentially mitigate ecological impacts of invasive species on native species and populations (Morissette et al. 2024). Moreover, environmental gradients can sometimes offer uninvaded refugia to native species if invasive species distribution and abundance are limited along the gradient, but the native species are not (i.e., physiological refugia; Chapman et al. 2002; Reid, Chapman, and Ricciardi 2013). However, across large spatial scales or strong environmental gradients, it is unclear whether physiological refugia result from generalist responses in physiological tolerance in native species (i.e., plasticity) or local adaptation of populations experiencing distinct environmental conditions. Likewise, invaders might impose additional divergent selection on native species because local populations that overlap with the invader could experience selection for anti‐predator traits (Strauss, Lau, and Carroll 2006; Brookes and Rochette 2007). Thus, although the interplay between adaptive responses and demographic characteristics is expected to be important for the resilience of native populations to biological invasions, the interaction between these factors is poorly understood.

While local adaptation could facilitate the coexistence of native species with an invasive predator (Melotto, Manenti, and Ficetola 2020), it could also restrict demographic processes that can be beneficial to the resilience of native species. Refuge populations can potentially serve as a source of demographic subsidy for individuals from invaded populations experiencing population decline (Foppen, Chardon, and Liefveld 2000; With, Schrott, and King 2006). Demographic rescue of populations occurs when immigrants from refuge populations provide individuals to bolster shrinking populations in invaded habitats (Hufbauer et al. 2015). Genetic rescue can additionally occur when migrants prevent the extinction of declining populations by increasing genetic diversity, thereby reducing inbreeding depression (Whiteley et al. 2015; Fitzpatrick et al. 2020). This positive outcome has generated considerable interest in conservation (Ralls et al. 2018; Hoffmann, Miller, and Weeks 2021; Willi et al. 2022) and is typically considered through the lens of individual translocation from diverse sources to inbred recipient populations (Fitzpatrick et al. 2016; Pavlova et al. 2017; Weeks et al. 2017). If this genetic variation includes adaptive alleles, then genetic rescue can also lead to an evolutionary rescue, that is, the avoidance of extinction via adaptation (Bell and Gonzalez 2011; Carlson, Cunningham, and Westley 2014; Hufbauer et al. 2015).

However, in wild populations connected through natural dispersal, local adaptation can reduce gene flow (Crispo 2008), as migrants have reduced fitness in divergent environments, through processes such as selection against migrants (Bolnick and Nosil 2007), which can result in patterns of isolation by environment (Wang and Bradburd 2014). Strong gene flow can conversely reduce the likelihood of adaptation due to genetic swamping (Tigano and Friesen 2016). Thus, there could be a tension between local adaptation of refuge and invaded populations and the potential for gene flow and its positive demographic/genetic rescue effects. It is, therefore, important to understand how different selective agents of adaptive change (to environmental conditions in the refuge vs. to the invader) interact with demographic processes to either facilitate or hinder the ability of a native species to avoid population decline from an invasive predator.

Genomic methods are increasingly used to understand species' and populations' responses to sudden environmental changes induced by anthropogenic activities such as invasive species (Stern and Lee 2020) and are an important tool for informing conservation (Willi et al. 2022; Bernatchez et al. 2024). These methods can enable the assessment of population connectivity, investigate demographic and genetic changes, and detect the potential for genetic adaptation (e.g., Marques et al. 2019). Reconstructing demographic changes can help assess potential population declines induced by invasive species. Additionally, inference of gene flow can identify the source and recipient populations in a metapopulation impacted by the invaders. Finally, assessing genetic adaptation can determine if source and recipient populations are divergent because of local adaptation (Cure et al. 2017), thus altering the likelihood of gene flow and demographic, genetic, or evolutionary rescue from genetically differentiated populations. Hence, knowledge of evolutionary forces, which can be elucidated through genomic tools, is critical for understanding the overall response of native species to the impact of biological invasions.

This study aimed to address the potential adaptation of a native gastropod (*Amnicola limosus*) to an invasive fish predator (*Neogobius melanostomus*, common name: round goby) along an environmental gradient in aqueous calcium in the Upper St. Lawrence River. We considered these processes in light of the potential for demographic and genetic rescue of invaded gastropod populations through gene flow from refuge populations, noting that *A. limosus* is not a species of conservation concern; the rescue effects considered here would occur through natural gene flow. Our objectives were to (1) test for the adaptation of *A. limosus* to the invasive predator and the low‐calcium habitats and (2) investigate if migrant gastropods could move from refuge populations to declining invaded populations (i.e., demographic rescue), which could also help maintain genetic diversity through gene flow (i.e., genetic rescue). We tested for local adaptation via a laboratory reciprocal transplant of wild *A. limosus* individuals to measure adult survival and fecundity, where snails were sourced from the St. Lawrence River (high‐calcium concentration, round goby present) or the Ottawa River (low‐calcium concentration, round goby absent) and exposed to home or transplant water treatment, along with (a factorially‐crossed treatment of round goby chemical cue [present vs. absent]). Genome scans based on whole‐genome data from wild populations were used to identify candidate SNPs underlying potential adaptation to low‐calcium habitats and to round goby predation. Finally, we applied population structure analyses and demographic modeling to document gene flow patterns, compare genetic diversity between invaded and refuge populations, and test for the effects of the invasion on effective population size *N*_e_.

For objective 1, we predicted that the gastropods would be locally adapted to calcium conditions and the presence of round gobies at the invaded sites. This would be indicated by (1) populations showing a home versus away advantage in life‐history traits in the transplant experiment, and (2) loci showing significant allele frequency differences in association with one or both covariables in the population genomics analysis. For objective 2, we predicted that we would observe a demographic signature of reduced effective population sizes and genetic diversity of *A. limosus* populations in invaded habitats, which would be consistent with a dramatic reduction in gastropod abundances that was observed in the Upper St. Lawrence River following round goby invasion in 2005 (Kipp et al. 2012). Moreover, in the case that we detected evidence of local adaptation to calcium conditions or the presence of predators, we predicted this may be accompanied by reduced gene flow between the habitat types, although we cannot directly ascribe a cause‐and‐effect relationship between these processes (e.g., reduced gene flow could be the result of weak water currents between these habitats). Our paper provides a rare empirical study to address how spatial heterogeneity in both abiotic conditions and invasive predator presence can interact with demographic processes to shape the response of a native species to biological invasion. It is important to not only consider ecological impacts of invasion but also the evolutionary and demographic responses of native species that can help foster invasive‐native species coexistence in freshwater ecosystems, which have been severely impacted by invasive species (Gallardo et al. 2016).

## 2 Material and Methods

### 2.1 Study System

Gastropods have been widely used to study adaptation in response to predation (Brookes and Rochette 2007; Hooks and Padilla 2021), with abiotic factors such as calcium concentration modulating this response through changes in shell morphology and behavior (Rundle et al. 2004; Bukowski and Auld 2014). As such, they are a useful biological study model for addressing evolutionary responses to biological invasions. *Amnicola limosus* is a small dominant freshwater gastropod species with a wide geographical distribution in the USA and Canada (www.gbif.org/species/5192461). This gastropod does not have a pelagic larval phase: egg masses are deposited on the substrate, and juveniles move from the substrate to the macro‐algal substrate (Pinel‐Alloul and Magnin 1973). Part of the range of *A. limosus* has been invaded by *Neogobius melanostomus* (common name: round goby), a molluscivorous fish, from the lower Great Lakes and running downstream throughout the Upper St. Lawrence River (Hickey and Fowlie 2005). *Amnicola limosus* is commonly found in the stomach contents of round gobies, and following the round goby invasion of Lake Saint‐Louis, *A. limosus* populations experienced a 0.5–1 mm reduction in shell size (Kipp et al. 2012). Because the mean gape size of the round goby is larger than the maximum size of *A. limosus*, round gobies do not have to crush the gastropod, which suggests that shell size reduction is likely to be due to reduced predation pressure on smaller and less visible individuals (round gobies are visual predators; Kipp et al. 2012). A considerable reduction in small gastropod abundance (down to 2%–5% of the original population size, with *A. limosus* being the most abundant species) and species richness in the Upper St. Lawrence River were also reported since the invasion of round gobies in this ecosystem in 2005 (Kipp et al. 2012). However, round gobies cannot tolerate low‐calcium concentrations (Baldwin et al. 2012; Iacarella and Ricciardi 2015), and have not invaded the Ottawa River at its junction with the Upper St. Lawrence River (Calcium concentrations below 22 mg/L; Sanderson, Derry, and Hendry 2021; Morissette et al. 2024). On the contrary, this low‐calcium concentration is not a physiological limit for *A. limosus* embryonic development (>1.1 mg/L; Shaw and Mackie 1990), and Pinel‐Alloul and Magnin (1973) showed that *A. limosus* was present in the Ottawa river before the invasion of round gobies, indicating that this species can tolerate the calcium concentration found in the Ottawa river. These calcium‐poor waters are thus acting as a refuge from round goby predation in this system (Astorg et al. 2021; Morissette et al. 2024). Calcium‐poor waters could potentially provide demographic subsidies for the native populations at invaded sites (e.g., amphipods; Derry, Kestrup, and Hendry 2013).

### 2.2 Study Sites, Sample, and Environmental Data Collection

**FIGURE 1:**
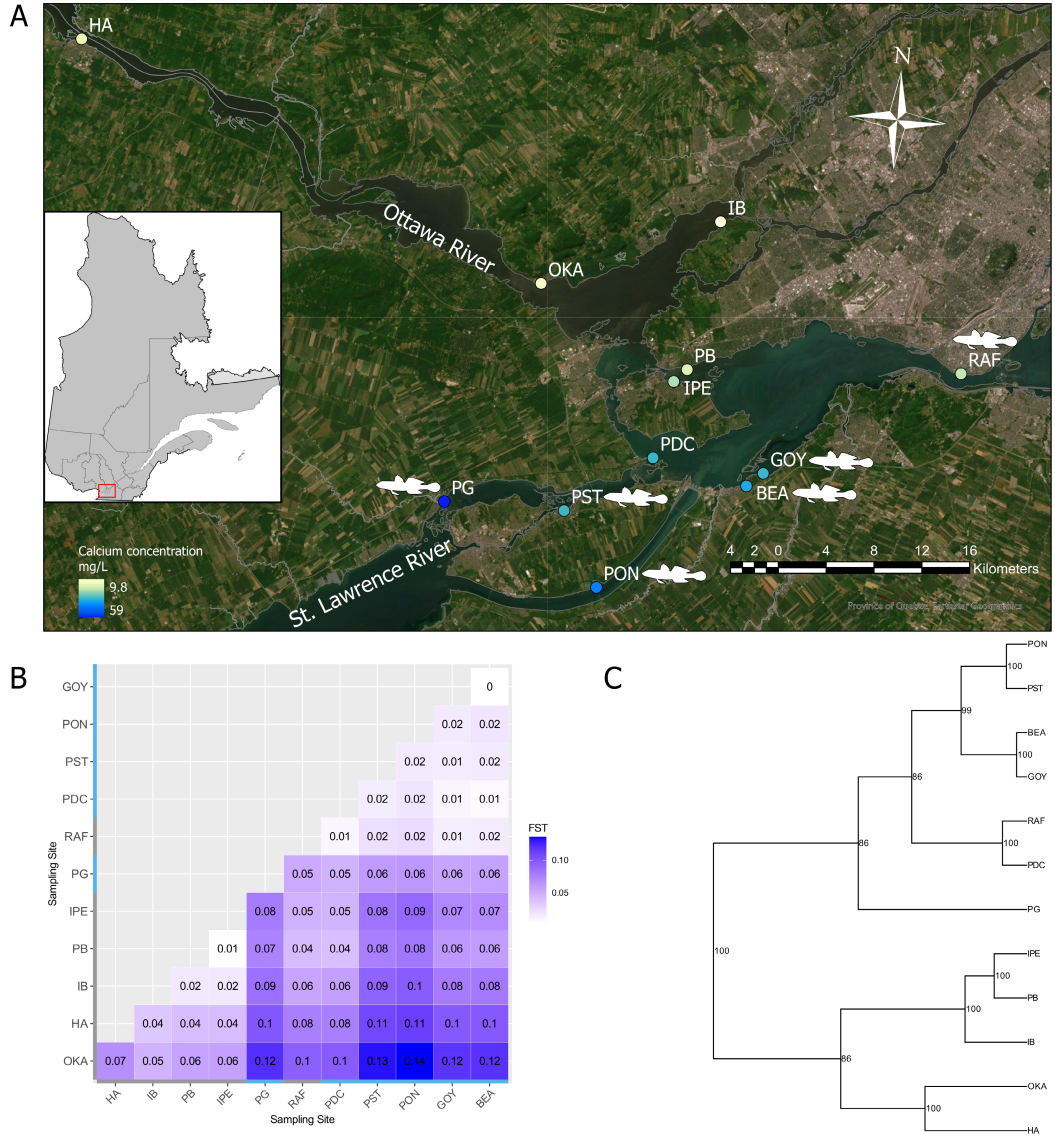
Study site locations in the Upper St. Lawrence River system and results of the population structure. (A) The study sites are located near the island of Montreal, QC, Canada (right of the map). Sites are colored based on water calcium concentration (mg/L). Sites with low calcium and round gobies absent are HA‐LCGA, OKA‐LCGA, IB‐LCGA, PB‐LCGA, and IPE‐LCGA while sites with high calcium and round gobies present are PG‐HCGP, PST‐HCGP, PON‐HCGP, BEA‐HCGP, and GOY‐HCGP. The two exceptions are RAF‐LCGP (low calcium and round gobies present) and PDC‐HCGA (wetland, high calcium, and round gobies absent), which have inverted patterns. (B) Pairwise *F*_ST_ matrix between the 12 study populations based on non‐outliers SNPs. The gray bars correspond to the Ottawa River populations (LCGA) and the blue bars to the St. Lawrence populations (HCGP), except for PDC (HCGA, wetland refuge) and RAF (LCGP). (C) Phylogenetic relationships between populations are shown as an UPGMA tree, with numbers indicating support for each node based on 1000 bootstraps.

Twelve study sites were located at the junction of the Ottawa River and the St. Lawrence River near Montreal, QC, Canada (Figure 1A). Ottawa River water is calcium‐poor (10–15 mg/L calcium), and St. Lawrence River water is comparatively calcium‐rich (30–40 mg/L) due to the different geological characteristics of their watersheds. These water masses join at the junction of two major river systems at Lake Saint Louis, a widening of the St. Lawrence River, but the calcium gradient persists between the north and south shores, and water masses are distinct (Vis, Cattaneo, and Hudon 1998). In 2005, round gobies invaded the upper St. Lawrence River and the southern shore of Lake Saint‐Louis, but not the calcium‐poor Ottawa River nor calcium‐poor sites on the north shore of Lake Saint‐Louis (Kipp and Ricciardi 2012).

We sampled 12 *A. limosus* populations from the study sites in this fluvial ecosystem (Figure 1A), with three populations fully in the Ottawa River, three fully in the St. Lawrence River, and six populations in the Lake St‐Louis, including three on the north shore and three on the south shore. The gastropod species identification was verified in the lab. We confirmed that our sampling locations corresponded to distinct population units with our population structure analyses (Figure 1B,C; Figure S1). Two populations in close proximity (2.3 km; BEA and GOY) showed low levels of differentiation (*F*_ST_ = 0.005), but the scaled covariance matrix Ω indicated that these two populations were indeed distinct (correlation coefficient between pairs of populations *ρ*_ij_ = 0.8, Figure S1). We coded populations collected in the Ottawa River water as LCGA (low‐calcium water and round gobies absent) and populations from the St. Lawrence River water as HCGP (high‐calcium water and round gobies present). Two populations had inverted patterns: RAF is calcium‐poor, but round gobies are present (LCGP), and PDC is calcium‐rich, but round gobies are absent (HCGA). It is noteworthy that PDC is located in a refuge habitat (wetland; Astorg et al. 2021) but is close to invaded sites (~8 km from the nearest invaded site). Field‐collected *A. limosus* gastropods were obtained near the shore via hand picking and brought back to the lab for further processing (DNA extractions and the common garden experiment) in June–October 2017. Round goby abundance was measured in the field between July and September 2017 on a single occasion at each site. For this, each site was sampled using three seine net passes. The seine net used for sampling near‐shore habitats was 9.14 m long by 1.8 m deep and 1/8 mesh on a 10 m distance. Round gobies were placed into bins and released after the three hauls. The geographic location and environmental characteristics of our sampling sites are detailed in Table S1. We measured dissolved oxygen (DO; mg/L), pH, water temperature (°C), and conductivity (μS/cm^2^) using a Professional Plus Model YSI multi‐parameter probe (model 10102030; Yellow Springs Inc.) at each study site in 2017 at the time of gastropod collection. On the same occasions, we collected water samples and analyzed them for calcium (Ca), total phosphorus (TP), total nitrogen (TN), as well as dissolved organic carbon (DOC) at the GRIL‐UQAM analytical lab (Appendix S1). Site‐specific invasion status by round goby (invaded/refuge) is defined by presence/absence (Table S1).

### 2.3 Reciprocal Transplant Experiment

**FIGURE 2:**
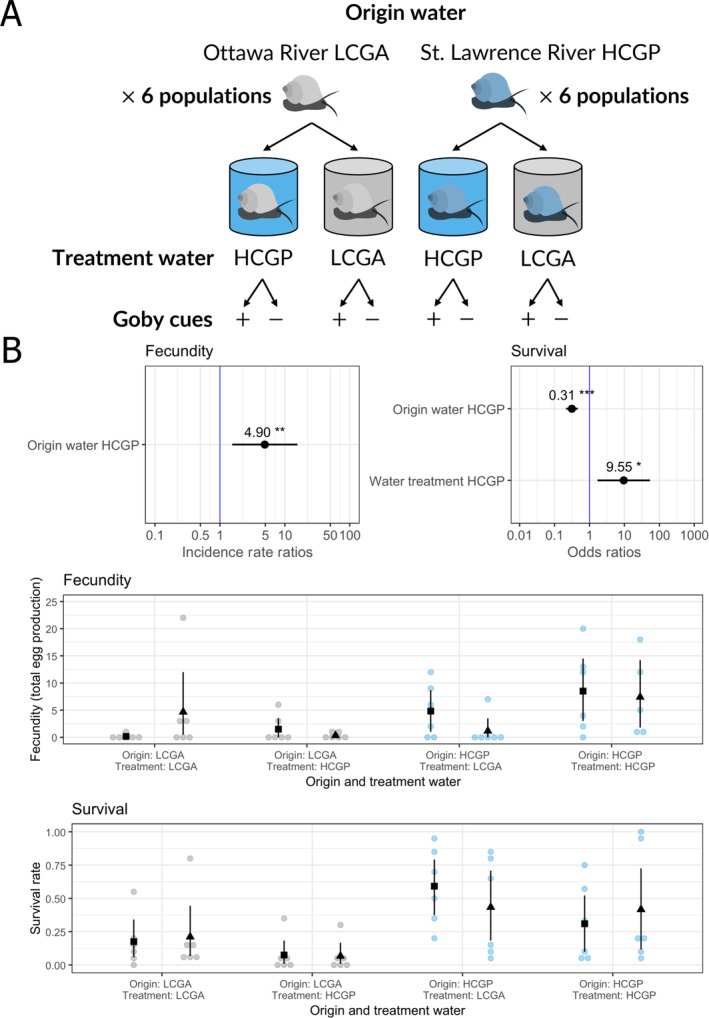
Experimental design of the laboratory reciprocal transplant experiment and results for the fecundity (total number of eggs produced) and survival of adult *Amnicola limosus* as a function of water treatment, origin water, and round goby cue treatment in the reciprocal transplant experiment. (A) Experimental design showing the two origin waters (Ottawa River Low Calcium/round Goby Absent LCGA in grey or St. Lawrence River High Calcium/round Goby Present HCGP in blue) with six replicate populations each, the water treatments (LCGA and HCGP) and the round goby cue treatment (+/–: With or without). (B) Top: Results of the converted coefficients of significant fixed effects (incident rate ratios and odds ratio) of the GLM and GLMM used to analyze fecundity and survival, respectively. Asterisks indicate threshold of *p*‐values: * for *p* < 0.05, ** for *p* < 0.01, *** for *p* < 0.001. Bottom: Raw data of the experiment, each dot represents a measurement for one population (origins in grey: LCGA, blue: HCGP), summarized by the mean for each treatment (black squares and triangles for treatments with or without round goby cues, respectively) and the 95% confidence interval around the mean (bootstrapping method).

We conducted a laboratory reciprocal transplant experiment at the Université du Québec à Montréal (UQAM) with field‐collected F_0_‐generation *A. limosus* to investigate the response of gastropods from populations experiencing different source habitat types (low calcium/refuge Ottawa River LCGA or high calcium/invaded St. Lawrence River HCGP) to home and transplant water (calcium‐poor water from the Ottawa River LCGA or calcium‐rich water from the St. Lawrence River HCGP), in the presence or absence of round goby cues (Figure 2A). The round goby cue treatment was used to test for predator effects on life‐history fitness components (adult survival and fecundity). *Amnicola limosus* snails that were involved in the experiment were mostly at adult or sub‐adult stages as we selected the largest individuals collected in the field and the dates of collection correspond to the presence of adult cohorts in the field (Pinel‐Alloul and Magnin 1973). Two additional water treatments were also tested: the artificial freshwater medium COMBO (Kilham et al. 1998), with and without the addition of calcium, to test for the specific effect of calcium (Ca) concentration on fitness components. The overall design was, therefore, a two (origin water: St. Lawrence River LCGA vs. Ottawa River HCGP) × four (treatment water from St. Lawrence HCGP vs. Ottawa River LCGA, COMBO growth media with/without Ca) × two (presence vs. absence of round goby cue) factorial experiment, with 12 replicates (corresponding to our sampling populations) per treatment combination. As the experiment was conducted within a single generation, we acknowledge that our reciprocal transplant experiment did not allow us to differentiate plastic versus genetic versus maternal effects on the measured traits.

We raised wild F_0_ individuals from the 12 populations in the laboratory for up to 73 days. Between 15 and 22 individuals (average: 19.6 ± 1.3) were initially placed in 250 mL plastic cups with river water and reared in growth chambers (Thermo Scientific Precision Model 818) at 18°C with a light:dark photoperiod cycle of 12:12 h. We fed *A. limosus* snails ad libitum with defrosted spinach every 2–3 days if needed or at each water change. Water in the water treatments was changed, and old spinach was removed every 3–4 days. For the round goby cue treatment, round gobies were kept in a 50‐L aquarium for 2 weeks before the experiment, set in a growth chamber at 18°C with a 12:12 h light. Round gobies were fed 3–4 times a week with flake fish food (TetraFin). The round goby cue treatment was added as 5 mL of water from the round goby aquarium per *A. limosus* culture at each water change (every 2–3 days), which represents 2% of the volume of the culture. The addition of water was done manually with a 30‐mL syringe. We recorded the survival of the adults and their fecundity (total number of eggs produced per individual) as response variables every 19 ± 13 days throughout the experiment, using high‐resolution stereomicroscopes (Olympus). However, due to the very low adult survival for all populations for the treatment testing the effects of calcium in growth media, we removed this comparison from further analyses (see Figure S2).

We analyzed fecundity (total number of eggs produced) and adult survival rates with a generalized linear model (GLM) and a generalized linear mixed effect model (GLMM) using the lme4 package in R, respectively. We modeled fecundity with a negative binomial distribution and adult survival with a binomial distribution and a logit link function. We checked the models for overdispersion using the overdisp_fun function from https://bbolker.github.io. We tested both models with and without the random effect of populations, using an AIC approach corrected for small sample size (AICc) and the ΔAIC criterion to evaluate the random effects (kept when ΔAIC > 2) with the R package bbmle. Likelihood ratio tests were used to evaluate the fixed effects for both the GLM and GLMM models. For the GLM model of fecundity, we checked for the influence of outliers on the model, by using both visual and quantitative diagnostics of the leverage and Cook's distance. We did not find a consistent effect of outliers on this model and thus did not remove outliers. Fixed effect coefficients and their confidence intervals were converted to incident rate ratios (fecundity) and odd ratios (adult survival) using an exponential function.

### 2.4 De Novo Genome Assembly and Pool‐Sequencing

As pool‐sequencing approaches require a reference genome (Schlötterer et al. 2014) to align the raw sequence reads and to conduct analyses that require information about the position of SNPs on the genome (e.g., to calculate diversity indices along the genome; Kofler et al. 2011), we conducted a de novo genome assembly for *A. limosus*. We extracted DNA from the tissue of one individual snail collected in 2017 using a standard Phenol Chloroform extraction method, after removing the shell and excising the mollusk guts to avoid contaminants. Briefly, tissue samples were placed in a digestion buffer containing proteinase K and digested at 55°C. DNA was then isolated using an isoamyl‐phenol‐chloroform solution, followed by ethanol precipitation. DNA quantity and quality were verified using a combination of different quality control methods: Qubit assay (Thermo Fisher Scientific Inc.), Tapestation (Agilent Inc.), and Femto Pulse (Agilent Inc.). Fragments longer than 1 kb were selected for further processing. Library preparation was performed using 10× Chromium Linked‐Read library kit (10× Genomics Inc.) and sequenced on three lanes of Illumina HiSeqX PE150 at Genome Quebec. A long‐read approach (10×) was selected for the sequencing as long‐read‐based assembly methods are more powerful at dealing with some issues (e.g., repeats, high GC content) arising in short reads‐based assemblies (Jung et al. 2020). We ran fastp v.0.23.4 on the three 10× paired‐end reads to obtain the insert size, using the ‐Q option to disable quality filtering. Fastp results showed two estimated insert size peaks at 175 and 270 bp. Reads were assembled with Supernova v.2.1.1. The assembled genome is 1,899,346,312 bp in length, with 815,134 scaffolds and a N50 of approximately 5 kb. We estimated the genome size with Jellyfish 2.3.0 by reading simultaneously the R1 reads of the three runs of 10× sequencing using the options ‐F 3, ‐m 21, and ‐s 2G. The resulting histogram was then processed with GenomeScope http://qb.cshl.edu/genomescope/ (Vurture et al. 2017), which yielded an estimated haploid genome length of 382,882,063 bp, with 2.25% of repeats and 4.72% of heterozygosity. This is much smaller than the assembled genome size (1,899,346,312 bp) due to fragmentation. We also used Benchmarking Universal Single‐Copy Orthologs BUSCO v5.2.2 (Manni et al. 2021) to assess gene completeness by searching for core mollusk orthologous genes, using the option ‐genome and the BUSCO.v4 lineage mollusca_odb10.2019‐11‐20. Most core genes were missing from our draft genome (74.7%), with only 19% complete core genes recovered (C: 19% [S: 18.1%, D: 0.9%], F: 6.3%, M: 74.7%, *n* = 5295, with C: complete single‐copy BUSCO genes, S: complete and single‐copy BUSCOs, D: complete and duplicated BUSCOs, F: fragmented BUSCOs, M: missing BUSCOs, *n*: total BUSCOs searched). We acknowledge that the draft assembly presented here is of low‐quality and completeness. However, the draft genome was sufficient for our population genomics goals, as we were not attempting to resolve the precise genetic architecture of specific traits (Savolainen, Lascoux, and Merilä 2013). For a similar application, see Brennan et al. (2022).

For the pooled sequencing, we extracted DNA from the tissues of 40 individuals per pool/population using the same standard Phenol Chloroform extraction method mentioned above. We quantified all samples using a Picogreen ds DNA assay (Thermo Fisher Scientific Inc.) on an Infinite 200 Nanoquant (Tecan Group Ltd). Samples were normalized to a dsDNA concentration of 15 ng/μL, re‐quantified, and pooled according to the sampling population. Thus, we created 12 pools of 40 individuals each at 15 ng/μL. Libraries were prepared with the NEB Ultra II kit for shotgun sequencing and sequenced on five lanes of HiSeq2500 125 bp pair‐ended at Genome Quebec. The number of reads sequenced per population was between 187 and 248 million paired‐end reads. We used the following formula to calculate the expected mean coverage: read length × number of reads/estimated haploid genome length. Given an estimated genome size of 382,882,063 bp, a read length of 125 bp, and 93.7–124.2 million single‐end reads sequenced, we calculated that our expected mean coverage was between 30 and 40×. We assessed the quality of our pool‐seq Illumina libraries with fastqc 0.11.5, from which we obtained a percentage of repeats between 18.4% and 39.7%. Pool‐seq presents some caveats as it offers limited information about linkage disequilibrium (Feder, Petrov, and Bergland 2012) and can lead to bias during the estimation of allele frequencies (Gautier et al. 2013). However, a large array of methods have been developed to account for such biases and accordingly, we have used Baypass and poolFreqDiff to detect outlier loci as well as poolfstat and popoolation to estimate genetic diversity indices (Kofler et al. 2011; Kofler, Pandey, and Schlötterer 2011; Schlötterer et al. 2014; Gautier 2015; Wiberg et al. 2017; Gautier et al. 2022).

### 2.5 Read Processing and SNPs Calling

We prepared the assembled reference genome of *A. limosus* by first indexing it with the Burrows‐Wheeler Aligner (BWA; Li and Durbin 2009) v0.7.17 and with Samtools faidx v1.12, and by creating a dictionary with Picard Tools v2.23.3. We then used a custom pipeline for pool‐seq quality processing, read alignment, and SNP discovery. We first trimmed reads with the function trim‐fastq.pl from popoolation v1.2.2 (Kofler et al. 2011) for a base quality of 20 and a minimum length of 50 bp, and assessed the quality of the trimmed reads with fastqc. We aligned trimmed reads to the reference genome with bwa‐mem v0.7.17. We filtered out ambiguously aligned reads with samtools v1.13 using a score of 20 and sorted bam files with samtools. We used samtools flagstat to find the percentage of Illumina reads aligned to the reference genome, which was on average 53.5% SD 4.0%. We obtained an mpileup file with samtools mpileup, then filtered SNPs with a minimum coverage of five across all populations. We converted the mpileup file to a sync file with Popoolation2 v1.10.03 (Kofler, Pandey, and Schlötterer 2011), with a quality score of 20. The sync file was then converted to a “pooldata” object with the poolfstat package in R (Hivert et al. 2018), using a haploid pool size of 80 for all populations, a minimum read count per base of two, a minimum coverage of five and a maximum of 300, a minimal minor allele frequency of 0.0125 (to remove singletons) and discarding indels. This pipeline retained 21,312,700 biallelic SNPs.

### 2.6 Detecting Genomic Signatures of Selection

To detect putative loci under selection, we used both outlier and environmental association analysis approaches with the hierarchical Bayesian models implemented in Baypass (Gautier 2015). Baypass is advantageous in the context of our study as it enables the detection of outlier SNPs after taking demographic history into account, thus avoiding the confounding effects of population structure. The core model estimates the scaled covariance matrix Ω of population allele frequencies, which summarizes population history. The scaled covariance matrix Ω is based on the deviation of population allele frequency from an average allele frequency calculated across populations, where populations that are more closely related tend to have more similar deviations (i.e., covariance) from the average (Coop et al. 2010). The scaled covariance matrix Ω is calculated based on the whole SNP dataset (non‐outliers and outliers) and is not related to pairwise *F*_ST_ except in some conditions (Coop et al. 2010). During the calculation of SNP‐specific statistics of differentiation (XtX and BF_is_ see below), this covariance is then explicitly accounted for through the scaled covariance matrix Ω (Gautier 2015). The full dataset was divided into 27 pseudo‐independent datasets to overcome computing limitations. The “pooldata” object from poolfstat was converted to the 27 sub‐dataset Baypass input files with the “thinning” subsampling method and sub‐sample size of 750,000 SNPs. We conducted the outlier analysis using the core model, to estimate the XtX statistic and associated *p*‐value under a *χ* ^2^ distribution with 12° of freedom (bilateral test, Baypass manual). The XtX statistic is similar to a SNP‐specific *F*_ST_ but corrects for covariance using the scaled covariance matrix Ω (Gautier 2015). We considered SNPs as outliers when their *p*‐value derived from the XtX estimator was <0.001. The shape of the histogram *p*‐values derived from the XtX statistics confirmed that they were well‐behaved (A peak close to 0 for loci putatively under selection and a uniform distribution between [0,1] for neutral loci; Figure S3B; François et al. 2016). The scaled covariance matrices Ω from the 27 sub‐datasets were compared visually to assess the concordance of the results, and then the statistics obtained for each sub‐dataset were combined (Baypass manual).

For the environmental association analysis, we opted for the standard model STD under the Importance Sampling approach in Baypass, in which the association between covariables and SNP allele frequencies is assessed independently for each covariable. If this model is not able to distinguish the effect of the calcium concentration from the effect of round goby presence/absence, we should observe a large overlap between outlier SNPs associated with each covariable (see the Appendix S1 for a more in‐depth explanation of the selection of the STD model). This model computes for each SNP its regression coefficient *β*_ik_ of the association between the SNP allele frequencies and a covariable to compare the model with association (*β*_ik_ ≠ 0) against the null model (*β*_ik_ = 0), from which a Bayes factor BF_is_ is derived. We selected two environmental covariables: invasion status (presence/absence of the round gobies) and calcium concentration. We also estimated the *C*_2_‐statistic with the STD model (Olazcuaga et al. 2021), which is more appropriate for binary variables and was used for the association with round goby presence/absence. We checked the Pearson correlation coefficient between covariables with the function pairs. Panel() in the package psych in R, which was *r* = 0.71 (slightly above the recommended threshold for the regression method of |*r*| < 0.7, Figure S4). For the calcium covariable, we ran three independent runs of the STD model with the random number ‐seed option, then computed the median BF_IS_ across runs to ensure the convergence of the MCMC results. To check for convergence of the independent runs, we calculated the Förstner and Moonen distance (FMD; Förstner and Moonen 2003) between the scaled covariance matrix Ω matrices from each sub‐dataset with the fmd.dist function in R (included in Baypass). Results were found to be convergent, with all FMD values <0.12. Covariables were all standardized to $\hat{\mu }$ = 0 and $\hat{\alpha^{2}}$ = 1, and we used default parameters for the MCMC analyses. For the calcium association, SNPs were considered significantly associated with a covariable when BF_is_ > 20 dB (Jeffrey's rule for “decisive evidence”; Gautier 2015). For the association with round goby presence/absence, we used the R package *q*‐value to correct for multiple hypothesis testing on the *p*‐values derived from the *C*_2_‐statistic and applied a False Discovery Rate of *α* = 0.01 as a *q*‐values cut‐off for outlier detection (Olazcuaga et al. 2021).

As a complementary analysis to investigate the potential for adaptation to the invasive predator and low‐calcium concentrations, we used poolFreqDiff (Wiberg et al. 2017) to identify outlier SNPs showing consistent allele frequency differences in the same direction between replicate populations contrasting the two calcium concentration (low vs. high) and round goby predation (absent vs. present) environment types. Note that with this approach, our aim was not to identify independent instances of parallel adaptation but rather detect genotype‐environment associations; consistent allele frequency differences could arise due to adaptation occurring in a shared recent ancestor. This method relies on modeling allele frequencies with a generalized linear model (GLM) and a quasibinomial error distribution, which should result in a uniform distribution of *p*‐values between [0,1] under the neutral (null) scenario (Wiberg et al. 2017). It also accounts for bias in allele frequency estimation (e.g., Gautier et al. 2013) by rescaling allele counts to an effective sample size *n*_eff_ (Feder, Petrov, and Bergland 2012). We ran the analysis separately for the two covariables as binary comparisons: invasion status (presence/absence) and calcium concentration (low <24.3 mg/L, high >34.3 mg/L). For the minimum read count per base, and the minimum and maximum coverage, we used the same values as for the poolfstat filtering, and we also rescaled the allele counts with *n*_eff_ and added one to zero count cells. To account for demography and genetic structure, we applied the empirical‐null hypothesis approach (François et al. 2016) to recalibrate *p*‐values based on a genomic inflation factor of λ = 0.85. We confirmed that recalibrated *p*‐values were well‐behaved based on the observed peak close to 0 and the uniform distribution between [0,1] (Figure S5; François et al. 2016). We then transformed the recalibrated *p*‐values into *q*‐values with the R package *q*‐value, and defined outliers if their *q*‐value was below the FDR *α* = 0.01.

We considered candidate SNPs for adaptation to low‐calcium concentration or to the round goby predation as SNPs that were in common between the environmental association analyses with Baypass and poolFredDiff and were uniquely associated with each covariable (Figure S6). We also excluded SNPs showing inconsistent allele frequency associations with environmental variables, which were considered false positives (*N* = 2 for the association with the calcium gradient and *N* = 1118 for the association with round goby predation). Note that the positive or negative associations with the covariables do not correspond to positive or negative selection, as the reference allele is chosen arbitrarily in poolfstat (and thus in Baypass).

### 2.7 Population Structure, Genetic Diversity, and Demography

We first estimated population structure with a genome‐wide pairwise *F*_ST_ matrix from the poolfstat package, using the same parameters as described above and removing the outlier SNPs detected with the Baypass and poolFredDiff analyses. We used the pairwise *F*_ST_ matrix to visualize phylogenetic relationships between populations with an UPGMA tree implemented in the R package ape and evaluated the support of nodes with bootstrapping (*N* = 1000). The pairwise *F*_ST_ matrix was also used to assess the potential for isolation by distance, using the relationship between the genetic distance (*F*_ST_/(1−*F*_ST_); Rousset 1997) and the log of the geographical distance (2D distribution of populations) with a Mantel test (9999 permutations) using the vegan package in R. Distances between sites were obtained by measuring paths between populations along the rivers (in m) with Google Earth v.10.38.0.0. Two outliers were present In the IBD analysis, but the relationship was still significant when these two data points were removed. We tested for isolation by environment, by calculating the environmental distance between population pairs using the squared Mahalanobis distance, determined from the calcium concentration and round goby presence/absence with the R package ecodist. We verified that there was no correlation between the environmental distance and geographic distance (non‐significant Mantel test with 9999 permutations: *r* ^2^ = 0.03, *p* = 0.20). Then we tested for a relationship between environmental distance and genetic distance as *F*_ST_/(1−*F*_ST_) with a Mantel test (9999 permutations). Finally, we compared the results of the pairwise *F*_ST_ matrix with the scaled covariance matrix Ω of population allele frequencies from the core model in Baypass, which summarizes some aspects of population history, though the scaled covariance matrix Ω is based on the complete dataset and thus includes outliers and putatively neutral SNPs.

To test if the round gobies impacted levels of genetic diversity in invaded *A. limosus* populations, we compared diversity indices between invaded and refuge populations. However, it should be noted that moderate bottlenecks do not necessarily trigger decreases in nucleotide diversity and heterozygosity (Adams and Edmands 2023), particularly in the initial generations after a bottleneck or in the presence of high levels of standing genetic variation or gene flow (Gompert et al. 2021). We obtained the observed heterozygosity from the poolfstat package and compared heterozygosity levels between round goby‐impacted and refuge populations with a *t*‐test after checking for the assumptions of normality (qqplot) and homoscedasticity (Bartlett test). We calculated genome‐wide diversity indices (Tajima's pi, Watterson theta, and Tajima's *D*) using popoolation (Kofler et al. 2011). First, we generated mpileup files for each population separately with samtools (Li et al. 2009) from the sorted. bam files output by the custom pipeline. Then we computed the genome‐wide diversity indices using non‐overlapping windows of 100 kb, a minimum coverage of 20 (as recommended in Kofler et al. 2011 except for Tajima's *D* with minimum coverage = 13, as the corrected estimator requires the pool size <3 minimum coverage), a minimum quality of 20, a minimum fraction covered of 0.05 and a pool size of 40. It should be noted that popoolation calculates the diversity indices along chromosomes; thus, due to the fragmentation of our draft genome, the diversity indices were calculated mostly among separate contigs and in windows <100 kb. The minimum number of SNPs per window across populations using this filter was 25. We used Hedge's *G* to detect a potential difference in the three diversity indices between the invaded and refuge populations.

We also investigated the demographic history of three population pairs using the diffusion approximation method implemented in δaδi (Gutenkunst et al. 2009). We aimed to detect a potential bottleneck in the invaded populations and to quantify the magnitude and direction of gene flow between the two habitat types (invaded and refuge). Due to the significant computational time required for these analyses, we selected a subset of populations from each habitat type for our models; we selected the populations PB‐LCGA, IPE‐LCGA, and PDC‐HCGA as refuges, paired with PG‐HCGP, BEA‐HCGP, and GOY‐HCGP as invaded populations, respectively. PDC‐HCGA was of particular interest as a high‐calcium population located in an uninvaded wetland (refuge). Our most complex model (Figure S7) has defined effective population sizes after the split (nu1 and nu2), followed by a bottleneck in both populations (modeling a scenario in which the round goby invasion impacted population abundance at the whole ecosystem scale) followed by exponential recovery in both populations. *T*_S_ is the scaled time between the split and the bottleneck and *T*_B_ is the scaled time between the bottleneck and present. Migration rates are asymmetric but constant through time after the split, with *m*_IR_ the migration from refuge to invaded populations and *m*_RI_ in the opposite direction. As we knew the time of the potential bottleneck (12 years before sampling with one generation per year), we set *T*_B_ as a fixed parameter. Because *T*_B_ is set as a fixed parameter, the parameter θ=4μL was an explicit parameter in the models that included a bottleneck. We defined *θ* with *μ* the mutation rate of 7.6 × 10^−9^ substitutions per site per year from the Caenograstropoda species *Nucella lamellose* (McGovern et al. 2010), and *L* the effective sequenced length, calculated as *L* ≈ total length of sequence analyzed × SNPs retained for use in dadi/total SNPs in analyzed sequence.

We investigated six additional non‐nested models: (a) bottleneck and growth only in the invaded population (constant *N*_e_ for the refuge population) with uneven migration, (b) only bottlenecks in both populations without recovery and uneven migration, (c) only bottleneck in the invaded population with uneven migration (constant *N*_e_ for the refuge population), (d) a simple population split at *T*_S_ with uneven migration, (e) a population split with symmetric migration and (f) a population split without migration (Figure S7). The default local optimizer was used on the log of parameters with random perturbation of the parameters to obtain a set of parameter values resulting in the highest composite likelihood. Optimization was conducted repeatedly until convergence was reached (i.e., three optimization runs with log‐likelihood within 1% of the best likelihood). One model in one population pair did not reach convergence after 30 optimization runs (bottleneck without recovery for the PDC‐HCGA and GOY‐HCGP population pair). Finally, we compared our seven models based on the differences in the likelihoods and plots of residuals of the models. We did not use AIC for model selection as it is not appropriate to compare composite likelihoods generated by dadi when SNPs are linked (Noskova et al. 2020) As we obtained unlikely results during the conversion of parameters in our best models, possibly due to imprecise mutation rates, we did not conduct parameter conversion. To obtain uncertainties on our parameters while accounting for the effect of linkage, we used bootstrapping and the Godambe Information Matrix approach (Coffman et al. 2016). For this, we generated 100 bootstrapped datasets with a chunk size of 1 × 10^5^ bp. Note that due to the draft genome fragmentation, the bootstrapping was executed between scaffolds of size usually smaller than 100 kb, which may have led to underestimated uncertainties.

To address the potential effect of using a pool‐seq approach on the variance in allele frequency estimates stemming from differences in coverage between pools (Gautier et al. 2013), we used a filter to obtain relatively homogenous coverage between our two selected populations/pools. From the initial SNPs dataset output by poolfstat (21,312,700 SNPs), we retained SNPs that fell within the 1st and 3rd quartiles of coverage in both populations (11–19× for PB‐LCGA and 10–18× for PG‐HCGP; 11–18× for IPE‐LCGA and 9–15× for BEA‐HCGP; 10–16× for PDC‐HCGA and 10–17× for GOY‐HCGP). We also filtered out SNPs that were detected as outliers (putatively under selection) in the Baypass (core and aux or STD models) and poolFreqDiff analyses and removed uninformative SNPs (fixed or lost in both populations). To accommodate for large computation time during the optimization, the datasets were thinned at random to retain a final dataset of ≈1 million SNPs per population pair. We used a custom script and the dadi_input_pools function from the genomalicious R package (Thia and Riginos 2019) with the “probs” parameter in the methodSFS option to transform allele frequency data into the SNP data format from δaδi. We used δaδi to infer the folded SFS as we did not have information on the ancestral allele state. Due to low confidence in the low‐frequency estimates, we also masked entries from 0 to 5 reads.

## 3 Results

### 3.1 Differences in Life‐History Traits Between Habitat Types

We found life‐history differences between the gastropod populations originating from the low‐calcium/ round goby absent and high‐calcium/ round goby present habitats, using fecundity and survival of adults as fitness components, as well as an effect of treatment water for survival (Figure 2B). For fecundity, the model with the random effect of population origin was not better than the model without (ΔAICc = 1.2), and only the origin water (Ottawa River: LCGA vs. St. Lawrence River: HCGP) effect was significant (*p* = 0.015). Even though the interaction between origin water and treatment water was not significant (*p* = 0.076), fecundity was higher for HCGP and LCGA populations in home water (13.30 SD 19.70 and 2.42 SD 6.27, respectively) than in transplant water (3.00 SD 4.33 and 0.92 SD 1.83, respectively). HCGP populations produced ≈5 times more eggs than LCGA populations overall, regardless of the treatment water (Figure 2B: 4.90, 95% CI [1.54–15.58]).

For adult survival, the model with a random effect of the population was better than without (ΔAIC = 250.7). The fixed effects of origin and treatment water were significant (*p* = 0.020 and *p* = 1.496 × 10^−9^, respectively), but their interaction and the round goby cue effect were not (*p* = 0.203 and *p* = 0.794, respectively). HCGP populations were 10 times more likely to survive compared to LCGA populations (Figure 2B: 9.55, 95% CI [1.70–53.73]). Adult survival was higher in home versus transplant water for individuals originating from LCGA habitats (0.19 SD 0.24 and 0.07 SD 12, respectively), but not for HCGP individuals (0.36 SD 0.36 and 0.51 SD 0.33, respectively). However, exposure to treatment water from the St. Lawrence River HCGP significantly lowered the odds of survival, with survival rates less than one‐third that of populations exposed to Ottawa River water LCGA (Figure 2B: 0.31, 95% CI [0.21–0.46]). There was considerable variation in survival rates between populations, as demonstrated by the significant effect of population on survival. Variation in survival among populations (random effect of the population of origin) did not depend on geographical location or habitat of origin (Figure S8): OKA‐LCGA, PG‐HCGP, and PON‐HCGP had significantly higher survival rates, while BEA‐HCGP, PDC‐HCGA, and PST‐HCGP had significantly lower survival rates.

### 3.2 Genomic Signatures of Local Adaptation to Round Goby Invasion and Water Calcium

Using the core model in Baypass as our outlier analysis, we found 226,794 outliers SNPs with *p* < 0.001 and either high or low XtX values, which represented ≈1.1% of the dataset (Figure S3A). Outlier SNPs with high XtX values can be interpreted as putatively under positive selection, while low XtX values indicate balancing selection (Gautier 2015). We also investigated the association of SNP allele frequencies with the selected environmental variables (invasion status and calcium concentration) using the STD model in Baypass. We found 778 outlier SNPs significantly associated with calcium concentration across the three replicate runs (BFis >20, ≈0.004% of the dataset) and 88,277 SNPs associated with the round goby presence/absence (*q* < 0.01, ≈0.4% of the dataset). Using the poolFreqDiff analysis with a FDR of 1%, we identified 54,285 outliers displaying consistent differences in allele frequency in the same direction between replicate populations in low versus high‐calcium habitats (0.3% of the dataset) and 23,651 outlier SNPs between the replicate populations in habitats with presence or absence of round goby (0.1% of the dataset; Figure S9). Of those calcium concentration outliers, 18 were in common between the Baypass STD model and the poolFreqDiff analysis, whereas 3324 of the predator status outliers were in common between the Baypass STD model (using the *C*_2_‐statistic) and the poolFreqDiff analysis (Figures S6 and S9). Most of the outliers were uniquely associated with a single covariable, with nine SNPs associated uniquely with the calcium gradient and 3320 SNPs with the round goby predation status (Figure S9). Overall, we found 1050 SNPs in common between the Baypass core and STD models, as well as 7009 SNPs in common between the Baypass STD model and the poolFreqDiff analyses including both the invasion status and calcium concentration (Figure S10).

**FIGURE 3:**
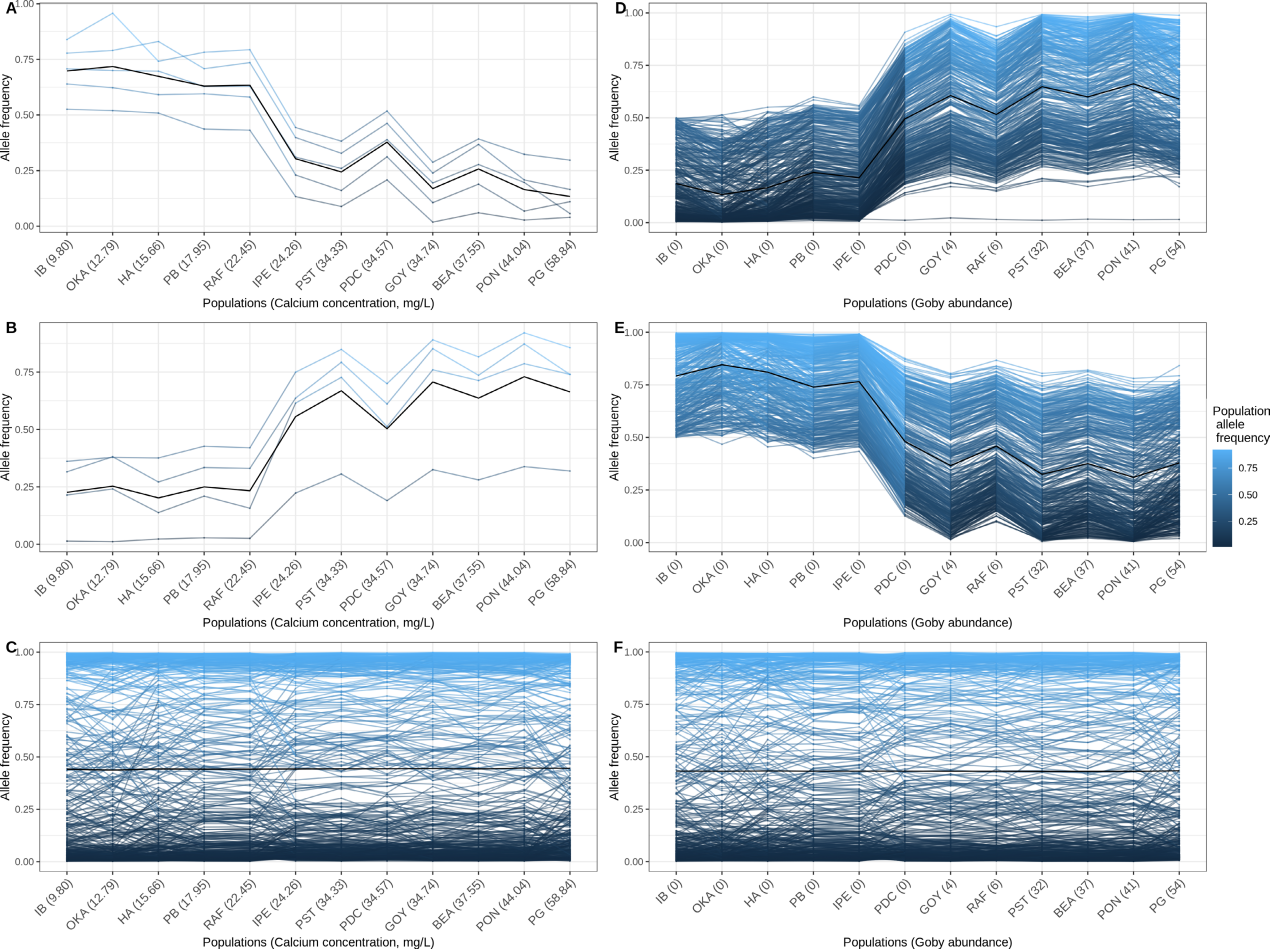
Allele frequency of candidate SNPs associated with the calcium gradient or with the round goby presence/absence, and of the non‐outliers SNPs. Note that we removed SNPs that showed inconsistent allele frequency changes compared to the other candidate SNPs considered false positives. The color scale indicates the allele frequency in a given population, and the black line is the average allele frequency change. (A) Candidate SNPs positively associated with the low‐calcium concentration. (B) Candidate SNPs negatively associated with the low‐calcium concentration. (C) Random subset of 400 non‐outlier SNPs non‐associated with the calcium gradient. (D) Candidates SNPs positively associated with the round goby presence (a random subset of 400 SNPs). (E) Candidates SNPs negatively associated with the round goby presence (a random subset of 400 SNPs). (F) Random subset of 400 non‐outlier SNPs non‐associated with the round goby presence/absence.

Candidate SNPs associated with calcium showed a baseline low (Figure 3A) or high allele frequency (Figure 3B) in high‐calcium populations (from PG to PST), then a sharp increase or decrease in allele frequency in population RAF, followed by a consistently high (Figure 3A) or low allele frequency (Figure 3B) in low‐calcium populations (from PB to IB). For the candidate SNPs for round goby predation adaptation, the baseline was a low (Figure 3D) or high allele frequency (Figure 3E) in populations where round gobies are absent (from IB to IPE), after which allele frequency had a sharp increase or decrease in population PDC, then stayed high (Figure 3D) or low (Figure 3E) in round goby‐impacted populations (from GOY to PG). Note that one of the populations with an inverted pattern of association between calcium concentration and round goby presence (RAF‐LCGP) had allele frequencies more similar to low‐calcium populations for candidate SNPs associated with the calcium gradient, while for the candidates associated with round goby presence/absence, the allele frequencies were in line with round goby present populations. The second population with an inverted pattern, PDC‐HCGA, had allele frequencies consistent with high‐calcium populations for the candidate SNPs associated with the calcium gradient, whereas the allele frequencies of round goby‐associated candidate SNPs were more similar to those of populations impacted by round goby predation.

### 3.3 Demographic and Genetic Effects of the Invasion

**FIGURE 4:**
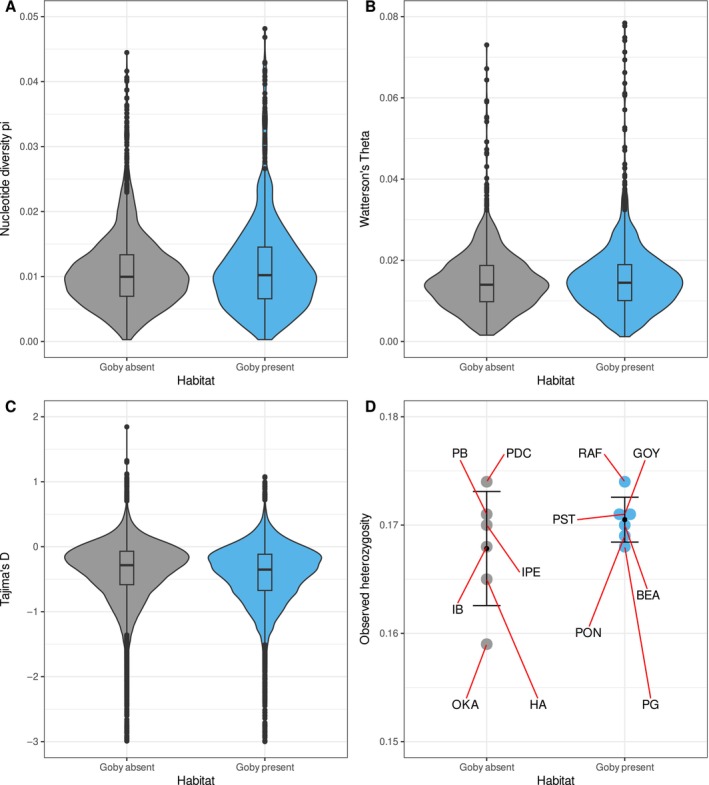
Genome‐wide diversity indices. (A) Violin plots of nucleotide diversity pi, (B) Watterson's Theta, (C) and Tajima's *D* according to predation level (round goby absent/present), with median and interquartile ranges shown in the box plot insert. (D) Observed heterozygosity per population, comparing round goby‐impacted and refuge populations.

Genome‐wide nucleotide diversity π was relatively high overall with 0.011 (SD 0.006) on average. Diversity was similar between the populations from the invaded habitats (0.012, SD 0.007) and populations from refuge habitats (0.011, SD 0.006), with a negligible effect size of habitat type (Figure 4A; Hedges' *g* = −0.104, 95% CI [−0.177, −0.031]). Estimates of *θ*_Watterson_ were identical between the two habitat types (Figure 4B; invaded populations: 0.015, SD 0.009; refuge populations: 0.015, SD 0.008), and the effect size of habitat was, therefore, negligible (Hedges' *g* = −0.074, 95% CI [−0.143, −0.005]). This resulted in a slightly negative overall Tajima's *D* (−0.38, SD 0.43), which was lower for invaded populations (−0.420, SD 0.440) than for refuge populations (−0.360 SD 0.431), but the effect size of the difference was negligible (Hedges' *g* = 0.137, 95% CI [0.121, 0.153], Figure 4C). Observed heterozygosity was not significantly different (*p* = 0.289, *t* = −1.153, df = 6.5; Figure 4D) between the populations from invaded (0.171, SD 0.003) and refuge sites (0.168, SD 0.003).

We found that the best demographic models were consistent with the known population declines in invaded habitats for two of the population pairs, with a significant association between invasion status and the effective population size (Table 1; Figure S11). The best model for PDC‐HCGA and GOY‐HCGP included a bottleneck without recovery only for the invaded population (Table 1). In the case of IPE‐LCGA and BEA‐HCGP, we did not detect a significant bottleneck, but the invaded population had an effective population size 40 times lower compared to the refuge and this difference was significant as indicated by the lack of overlap between the confidence intervals on the parameters nu1 and nu2 (Table S2). Finally, results were less clear‐cut for the pair PB‐LCGA (refuge) and PG‐HCGP (invaded): the best model included bottlenecks in both populations, but the inferred parameters suggested that only the invaded population showed *N*_e_ recovery (non‐significant increase between nu2B and nu2F, Table S2), which would correspond to an alternative model that we did not test (bottlenecks in both population but *N*_e_ recovery only in the invaded population).

**TABLE 1:** Results of the demographic models investigated with dadi for the three population pairs.

Model	*k*	Composite log‐likelihood per population pair		
		PG‐HCGP/PB‐LCGA	BEA‐HCGP/IPE‐LCGA	GOY‐HCGP/PDC‐HCGA
Bottleneck + growth two populations	10	−10,380	−12,816	−11,447
Bottleneck + growth invaded population	8	−10,736	−12,485	−11,846
Bottleneck two populations	8	−11,017	−12,884	−17,494
Bottleneck invaded population	7	−10,771	−12,334	−10,177
Split + uneven migration	5	−10,615	−11,669	−21,798
Split + even migration	4	−10,777	−12,257	−10,659
Split no migration	3	−24,606	−29,842	−21,809

*Note:* Three population pairs were selected: PG‐HCGP (invaded) with PB‐LCGA (refuge), BEA‐HCGP (invaded) with IPE‐LCGA (refuge), and GOY‐HCGP (invaded) with PDC‐HCGA (refuge). Models are presented according to their log‐likelihood, with the number of parameters in each model (*k*). For each population pair, the best model based on composite log‐likelihood is underlined.

### 3.4 Isolation by Environment and Variable Gene Flow Between Habitat Types

Using putatively neutral SNPs, we found that populations clustered by the presence/absence of round gobies (Figure 1B,C), except for PDC‐HCGA, which clustered with the invaded populations. The clusters (PON‐HCGP, PST‐HCGP, BEA‐HCGP, GOY‐HCGP, PG‐HCGP, RAF‐LCGP, and PDC‐HCGA vs. IPE‐LCGA, PB‐LCGA, IB‐LCGA, OKA‐LCGA and HA‐LCGA) had lower pairwise *F*_ST_ values within clusters than between them. This population structure was concordant between the pairwise *F*_ST_ matrix (Figure 1B, neutral SNPs only) and the scaled covariance matrix Ω (Figure S1, includes putatively neutral and outlier SNPs). Our results from the scaled covariance Ω matrix of population allele frequencies inferred with Baypass indicate that there was a positive covariance in allele frequencies within clusters and negative covariances between clusters (Figure S1). Population structure was also explained by isolation by distance and by the environment (Figure S12), as the positive correlation between the genetic distance *F*_ST_/(1−*F*_ST_) and the log of the geographic distance or the Mahalanobis were significant (Mantel test, 9999 permutations: *p* = 0.0007, *r* ^2^ = 0.285 and *p* = 0.044, *r* ^2^ = 0.078, respectively). We found low but significant asymmetric gene flow in most cases with the scaled migration rates 0.05 < 2*N*_e_m < 5.5 (Table S2). However, gene flow was non‐significant (2*N*_e_m < 0.05) from the low‐calcium refuge populations toward the high‐calcium‐invaded populations (IPE‐LCGA toward BEA‐HCGP and PB‐LCGA toward PG‐HCGP). For these two population pairs, the lower migration rates were in line with higher values of pairwise *F*_ST_ and time since the population split (Figure 1C; Table S2). Finally, the population pair GOY‐HCGP and PDC‐HCGA (wetland refuge) had much higher migration rates in both directions compared to the other population pairs, consistent with lower pairwise *F*_ST_ and these two populations belonging to the same cluster (Table S2; Figure 1C,D; Figure S1).

## 4 Discussion

We investigated the interaction between adaptation and demography to gain insight into the persistence of a native gastropod (*Amnicola limosus*) following approximately 12 years of exposure to an invasive predator, the round goby, at invaded sites in the Upper St. Lawrence River. Our genomic results are consistent with *A. limosus* undergoing local adaptation to the invasion in the span of ≤12 generations, with candidate SNPs showing a unique association with the presence/absence of round goby predators. These results were strengthened by the laboratory reciprocal transplant experiment, where populations originating from high‐calcium/round goby present habitats showed higher fecundity in home versus transplant treatment. They also had higher fitness overall (fecundity and adult survival) than populations from low‐calcium/round goby absent habitats regardless of the water treatment. We also found evidence for adaptation to differences in water calcium over the longer geological history of the ecosystem, suggested by the unique association of outlier SNPs with the calcium gradient, and the higher fitness of populations originating from the low‐calcium habitats in home and transplant water treatments. Despite evidence of local adaptation in invaded populations, they are experiencing a demographic decline. While these results could imply that the low‐calcium refuge populations have the potential to provide migrants and generate demographic and genetic rescue of invaded populations, this hypothesis was not supported. We detected restricted gene flow and strong population structure between the refuge and invaded populations. Moreover, we found evidence suggesting that adult *A. limosus* originating from low‐calcium refuges have low fitness overall for the life‐history traits measured (fecundity and adult survival) across all water treatments (home and transplant water) in the laboratory reciprocal transplant experiment. Therefore, despite the current persistence of native *A. limosus* gastropods in the Upper St. Lawrence River system following the invasion by round gobies, this native gastropod could become vulnerable due to reductions in effective population sizes and limited potential for genetic rescue of impacted populations by the populations in physiological refugia. Knowledge of evolutionary and demographic processes is crucial for our understanding of how native species will respond to biological invasion and the mechanisms that facilitate or inhibit their coexistence with invasive species.

### 4.1 Adaptive Responses to Round Goby Invasion and Water Calcium Levels

Our results from the reciprocal transplant experiment give insight into potential adaptive responses in life‐history traits between HCGP and LCGA populations to divergent calcium concentrations and round goby predation regimes (Figure 2). Indeed, we found important differences in life‐history traits (fecundity and survival of adults as fitness components) between the populations originating from the two habitat types. Both fecundity and survival were higher in the HCGP populations than in the refuge LCGA populations, regardless of water treatment. The potential for local adaptation to the calcium gradient is suggested by the significant water treatment effect on adult survival, and by a slight home advantage in fecundity for populations from both habitats in their origin water versus transplant water (water treatment LCGA vs. HCGP), although the interaction between origin and treatment water was not significant. Our genomic results support the idea that the life‐history differences observed between the two population types (LCGA and HCGP) could be at least partially explained by adaptive genetic differences between populations from the two environments (round goby present or low‐calcium).

While our laboratory reciprocal transplant experiment and genomic results could indicate that *A. limosus* responded to round goby invasion through shifts in life‐history traits, populations from the low‐calcium refuge habitats also showed low fitness across treatment water and round goby cue treatments. This could occur through a trade‐off of adaptation to low‐calcium water, as intracellular transport of calcium is energetically costly (Clark et al. 2020). In addition, individuals from the Ottawa River might allocate more resources toward calcium transport and be less able to invest in life‐history traits such as reproduction. Maternal effects could have also carried over from the less favorable low‐calcium environment into the laboratory experiment, as maternal effects can have negative consequences on offspring fitness (Marshall and Uller 2007) and we did not control for maternal effects in our experiment. Adult survival rates between populations varied widely, especially for the HCGP populations. This could reflect potential local (mal)adaptation to other biotic or abiotic parameters that we did not consider in the present study (e.g., temperature, substrate, nutrient availability, dissolved organic carbon concentration, and food quality).

### 4.2 Genomic Signatures of Local Adaptation to Round Goby Invasion and low Water Calcium

Our genomic data (population structure, Figure 1B,C and environmental association analyses, Figure 3; Figure S6) provide general evidence for local adaptation to the two distinct environment types in *A. limosus*, that is, low calcium/round goby absent and high calcium/round goby present. The exceptions were for two populations (RAF‐LCGP and PDC‐HCGA) that experienced inverse conditions for selection than the other sampled populations, both clustering with the invaded populations. Note that RAF‐LCGP is located on the north shore of Lake Saint Louis, where the water masses from the St. Lawrence River and the Ottawa River are still distinct (Vis, Cattaneo, and Hudon 1998), and is, therefore, located on the path of water currents originating from the Ottawa River. For this population, the results thus support strong selection from round goby predation even under lower calcium conditions, which are less optimal environmental conditions for round goby feeding and performance (Iacarella and Ricciardi 2015). For PDC‐HCGA, the results suggest strong migration from adjacent invaded sites, which was confirmed by our demographic analyses (see below). PDC‐HCGA itself remained uninvaded despite higher water calcium concentrations, likely because the site was located within a wetland, which provides less optimal conditions for round goby establishment due to the substrate properties (Astorg et al. 2021).

Our EA analyses with Baypass and poolFreqDiff potentially allowed us to disentangle the signals of the two putative selective pressures (i.e., the effect of selection from round goby predation at invaded sites and low‐calcium levels at refuge sites), even though invasion status and calcium concentration were strongly correlated. We found SNPs uniquely associated with invasion status and calcium concentration, which can be interpreted as signatures of local adaptation to predation by the round goby fish, and to the more limiting calcium concentrations at refuge sites. However, a more comprehensive understanding of the relative roles of these distinct selection mechanisms on genomic variation will require functional validation and ideally a different sampling design that includes additional sites with less correlation between these two environmental factors. Due to the unavailability of an annotated reference genome for *A. limosus* or a closely related species, we were unable to investigate putative physiological functions of the SNPs showing significant differentiation between environment types. Adaptation to calcium likely involves different functions from adaptation to predation. Differences in calcium concentrations between the water masses from the two rivers are related to the geological characteristics of the river watersheds and, therefore, represent environmental differences over the long evolutionary history of this species in the St. Lawrence River. On the other hand, predation from the invasive round goby on mollusks is a recent and novel stressor in the St. Lawrence River. Putative physiological functions that would be worth investigating in future studies include transmembrane calcium transport and biomineralization pathways that might be involved in adaptation to low‐calcium concentration (Clark et al. 2020), as well as shell development regulatory genes that could play a role in the evolution of smaller‐sized shells at maturity, which has been observed in populations subject to round goby predation (Kipp et al. 2012; Johnson, Fogel, and Lambert 2019).

### 4.3 Demographic and Genetic Effects of the Invasion

Given prior findings showing a decline in gastropod population abundance following invasion by round gobies, we hypothesized that invaded populations might suffer from population bottlenecks and reduced genetic diversity. However, we did not find a negative effect of the invasion on genetic diversity (Figure 4), with high levels of nucleotide diversity found in all populations. Our demographic analysis revealed the occurrence of genetic bottlenecks or significant reductions in *N*_e_ in invaded populations, and also in one refuge population (Table 1; Table S2). This reduction in *N*_e_ was only followed by recovery in one of the cases, hinting at a possible case of genetic rescue (i.e., due to an increase in genetic diversity), although the results must be interpreted with caution as there was considerable uncertainty around the parameter estimates. The source of migrants that could potentially be generating a rescue is also presently unknown and is unlikely to be due to the migration from the low‐calcium physiological refugia populations as the gene flow in this direction was non‐significant. Further validation of the potential for genetic rescue would require obtaining census data and collecting new genomic samples targeted at identifying the potential source populations and quantifying the level of hybridization in recipient‐invaded populations (Fitzpatrick et al. 2020). The effective population size reductions did not trigger major declines in genetic diversity levels (Figure 4), which could indicate that the bottlenecks were moderate (Adams and Edmands 2023) and that the high levels of standing genetic variation present have buffered the impacts of these bottlenecks. In the present study, the low but significant gene flow (0.1 < 4*N*_e_m < 11; Hämälä, Mattila, and Savolainen 2018) within habitats could likewise have contributed to the maintenance of genetic diversity, as suggested by the gene flow estimates detected between PDC‐HCGA and GOY‐HCGP populations and the lower *F*_ST_ within clusters (Table S2; Figure 1B). Similar gene flow rates as observed here have been shown to be sufficient to maintain genetic diversity despite low effective population size (Gompert et al. 2021).

### 4.4 Interaction Between Population Structure and Demographic/Genetic Rescue

A core goal of this study was to determine if local adaptation could interact with demographic and genetic rescue. We found relatively low or non‐significant levels of gene flow between the populations from the two habitat types (from LCGA refugia to HCGP populations and inversely), but high gene flow within habitat types (Table S2). Thus, our analyses of gene flow indicate that refuge populations in low‐calcium habitats do not provide migrants to invaded populations, and this is further supported by the significant pattern of isolation by environment (Wang and Bradburd 2014). This pattern could be generated by selection against migrants (Nosil, Egan, and Funk 2008; Orsini et al. 2013; Tigano and Friesen 2016) related to calcium limitation and round goby predation. Individuals from the LCGA populations had lower fitness overall compared to HCGP populations and thus might have low reproductive output and survival in invaded habitats. Given that round goby predation has been shown to cause a decrease in shell sizes at maturity (Kipp et al. 2012), LCGA individuals might be also more vulnerable to predation than HCGP individuals if they are more conspicuous due to larger shell sizes. Similarly, hybrids might be selected against if intermediate phenotypes have lower fitness in their local environment (Thompson et al. 2022). In addition, because individuals from refuge populations have lower reproductive output and survival, they provide a more limited demographic subsidy to invaded populations. This will depend on the magnitude of the relative fitness difference between source and recipient populations (i.e., how detrimental migrant alleles will be in the recipient populations; Bolnick and Nosil 2007). The recent adaptation to round goby predation might also have reinforced the effect of isolation by environment from the adaptation to low calcium. Additionally, the clustering of LCGA and HCGP populations could result from isolation by distance, which was significant, implying some restrictions to gene flow in this system, as we confirmed with our demographic modeling. The divergent selection could thus be a cause or a consequence of population structuring between the two habitat types (low vs. high calcium). Importantly, these apparent patterns of divergent selection are unlikely to be an artifact of population structure, as our genome scan approaches take this effect into account, and they are also supported by the results of our laboratory reciprocal transplant experiment. The strong population structure differentiating low‐calcium and high‐calcium populations is potentially caused by a combination of local adaptation, geographic distance, and migration patterns, thereby limiting the potential of low‐calcium physiological refugia to provide migrants necessary for both the demographic and genetic rescue of invaded populations.

In contrast to the low gene flow found between LCGA and HCGP populations, gene flow was relatively high between the wetland refuge PDC‐HCGA and the invaded population GOY‐HCGP, although it did not result in *N*_e_ recovery from the bottleneck in the latter (Table S2). Wetland habitats provide refuge from round goby predation by reducing their abundance at a local scale and are known to enhance fish and macroinvertebrate diversity (Astorg et al. 2021; Morissette et al. 2024). Their role as a refuge has also been previously recognized in other invaded systems (Reid, Chapman, and Ricciardi 2013). Given the prevalence of wetlands in the Upper St. Laurence River (Morissette et al. 2024), wetland refugia with high‐calcium concentration thus have the potential to provide migrants to invaded sites, particularly due to the lower adaptive divergence between these populations. This suggests that migration of individuals from larger wetland refuge populations might be providing not only a demographic subsidy (demographic rescue; Hufbauer et al. 2015) but could also replenish the genetic diversity if it was lost due to population declines in invaded populations (genetic rescue; Whiteley et al. 2015). The role of wetlands as a refuge and their potential implication in the demographic and genetic rescue of invaded gastropod populations, therefore, warrants further investigation.

Based on our demographic modeling and population structure results (Table S2; Figure 1B,C), populations from high‐calcium habitats are more likely to be exchanging migrants between them rather than with populations from low‐calcium refuge habitats, which appears to be sufficient for preserving genetic diversity. However, this beneficial effect of gene flow might have limitations as shown by the absence of effective population size recovery in two out of three populations for which we detected a bottleneck (Table 1). This is particularly important because strong selection such as that detected in the invaded populations can also lead to reduced population sizes and drift, with negative effects on population fitness (Falk, Parent, and Bolnick 2012). The net outcome of this conflict between local adaptation and genetic rescue will thus depend on the severity of population decline in recipient populations (Hufbauer et al. 2015), the extent of adaptive differentiation between populations in each habitat type, and the rate of immigration from high‐calcium wetland populations.

### 4.5 Implications: Potential Limitations of Genetic Rescue During Population Management

This study documents the impact of an aquatic invasive predator on evolutionary and demographic processes in a native prey species. Evaluating the potential evolutionary impacts of invasive species on native species is important because they can lead to surprising, unforeseen negative effects, such as the disruption of existing local adaptation (Melotto, Manenti, and Ficetola 2020). Genetic rescue has been proposed as a valuable tool for the conservation of small, isolated populations through translocations (Whiteley et al. 2015; Ralls et al. 2018). The present study highlights a case from natural, unmanaged populations where the potential for genetic rescue from physiological refugia is potentially limited by adaptive divergence and population structure that restricts gene flow between refuges and impacted populations. This implies that the presence of physiological refugia will not necessarily translate into the demographic or genetic rescue of imperiled populations, if strong genetic differentiation exists between refuge and recipient populations, for example stemming from isolation by environment. It thus reiterates the importance of considering the local (mal)adaptation of donor and recipient populations during managed introductions that aim to produce genetic rescue (Hoffmann, Miller, and Weeks 2021).

## Conflicts of Interest

The authors declare no conflicts of interest.

## Supporting information


Appendix S1.

## Data Availability

The raw sequencing reads have been deposited in the National Center for Biotechnology Information Sequence Read Archive SRA repository (BioProject PRJNA1035459) and the accompanying metadata are also stored in the SRA (BioProject PRJNA1035459), using the Eukaryotic water MIxS package. The draft genome of Amnicola limosus has been deposited on the NCBI Genome database (BioProject PRJNA1136450) and is currently being processed. The scripts and input data have been uploaded to the Dryad Digital Repository (https://doi.org/10.5061/dryad.rxwdbrvjq).
