# Supplementary material for: Limited Migration From Physiological Refugia Constrains the Rescue of Native Gastropods Facing an Invasive Predator
Source: Evol Appl. 2024 Oct 21;17(10):e70004. doi: 10.1111/eva.70004 (PMC11493756; doi:10.1111/eva.70004)
Supplement: Supplementary file 1 — Appendix S1. [file EVA-17-e70004-s001.docx]

**Supplementary material**

# Limited migration from physiological refugia constrain the rescue of native gastropod facing an invasive predator

**Supplementary Methods: Analytical methods for water chemistry**

We collected water samples to quantify dissolved calcium (Ca; mgL-1), total phosphorous (TP; µgL-1), total nitrogen (TN; mgL-1), and dissolved organic carbon (DOC; mgL-1). TP, TN, and DOC samples were analyzed at the GRIL- Université du Québec à Montréal (UQAM) analytical laboratory. Water calcium samples were analyzed with a Thermo ICAP-6300 Inductively Coupled Argon Plasma - Optical Emission Spectrometer (ICP-OES) following protocols described by US EPA (1994) at the University of Alberta Biogeochemical Analytical Service Laboratory (U of A – BASL; Edmonton, Alberta, Canada). Total Phosphorus (TP) was measured spectrophotometrically on the same machine by the molybdenum blue method after persulfate digestion (Griesbach and Peters, 1991). Total Nitrogen (TN) was analyzed with a continuous flow analyzer (OI Analytical Flow Solution 3100 ©) using an alkaline persulfate digestion method, coupled with a cadmium reactor, following a standard protocol (Patton and Kryskalla, 2003). DOC concentrations of 0.45 μm filtered samples (surfactant-free membrane filters) were analyzed with an OI Analytical Aurora 1030W TOC Analyzer (https://www.oico.com/1030W) using a persulfate oxidation method at the GRIL- Université du Québec à Montréal (UQAM) analytical laboratory.

EPA 1994. Method 200.7, Revision 4.4: Determination of Metals and Trace Elements in Water and Wastes by Inductively Coupled Plasma-Atomic Emission Spectrometry. Environmental Monitoring Systems Laboratory Office of Research and Development, U.S. Environmental Protection Agency, Cincinnati, Ohio 45268.

Griesbach S.J. & Peters R.H. (1991) The effects of analytical variations on estimates of phosphorus concentration in surface waters. Lake Reservoir Management, 7, 97–106.

Patton CJ, Kryskalla JR. Methods of analysis by the US Geological Survey National Water Quality Laboratory: Evaluation of alkaline persulfate digestion as an alternative to kjeldahl digestion for determination of total and dissolved nitrogen and phosphorus in water: US Department of the Interior, US Geological Survey; 2003.

**Supplementary Methods: Justification of the choice of model for the environmental association analysis with Baypass.**

On the recommendation of Baypass author Mathieu Gauthier, we used the standard covariate (STD) model in Baypass to better understand the role of these two co-variables. This model estimates association to each covariable independently (no allele frequency correction), which we combined with the contrast statistic C2 on the goby covariable (which is more appropriate for binary covariables). We obtain very little overlap in SNP outliers between the covariables based on the BFis (calcium conc.) and the C-statistic (goby presence/absence). We believe this reflects the reality of our dataset as we also obtained similar results when analyzing each covariable independently with poolFreqDiff. Our SNP dataset is very large (X SNPs) and only a small proportion of SNPs have a significant association with either variable (0.004% for calcium concentration and 0.4% for predator status with the Baypass STD model). As such, despite the covariables being highly correlated, because they are not perfectly correlated, the likelihood of these calcium-associated and predator-associated SNPs overlapping is relatively low. This lack of overlap makes it feasible to use the allele frequency patterns observed in the candidate SNPs to distinguish the independent effects of each covariable. However, we would like to emphasize that our central goal is to determine if the populations are locally adapted, and not necessarily to distinguish the precise mechanisms of selection. We recognize that our sampling design is not ideal for the latter.

**Table S1: Environmental characteristics of the study sites.** Ca: calcium concentration, TP: total phosphorus, TN: total nitrogen, DOC: dissolved organic carbon, Temp: water temperature, DO: dissolved oxygen, Cond: conductivity, Alk: alkalinity.

| Site name | Latitude | Longitude | Goby abund. | Time since invasion  (years) | Ca (mg/L) | TP (ug/L) | TN (ppm) | DOC (mg/L) | Temp (C) | DO (%) | Cond. | Alk. | pH |
| --- | --- | --- | --- | --- | --- | --- | --- | --- | --- | --- | --- | --- | --- |
| RAF-LCGP | 45.415387 | -73.633328 | 6 | <12 | 22.45 | 35.28 | 0.462 | 7.37 | 18.4 | 96.0 | 182.8 | 118.95 | 7.76 |
| PST-HCGP | 45.290666 | -74.044296 | 32 | 13-16 | 34.33 | 14.54 | 0.338 | 8.95 | 22.9 | 120.7 | 313.8 | 204.10 | 8.60 |
| PON-HCGP | 45.234976 | -74.003697 | 41 | 13-16 | 44.04 | 18.59 | 0.411 | 3.64 | 21.3 | 98.5 | 311.2 | 202.15 | 8.28 |
| PG-HCGP | 45.290416 | -74.172167 | 54 | 13-16 | 58.84 | 97.32 | 1.594 | 17.10 | 21.9 | 137.2 | 367.0 | 238.55 | 8.50 |
| PDC-HCGA | 45.335278 | -73.954167 | 0 | Uninvaded | 34.57 | 53.91 | 0.496 | 28.79 | 18.9 | 98.0 | 300.3 | 195.00 | 8.40 |
| PB-LCGA | 45.403333 | -73.925000 | 0 | Uninvaded | 17.95 | 44.12 | 0.660 | 21.95 | 24.0 | 98.4 | 130.3 | 84.50 | 7.81 |
| OKA-LCGA | 45.459444 | -74.087500 | 0 | Uninvaded | 12.79 | 76.67 | 0.765 | 13.52 | 22.9 | 106.4 | 115.2 | 74.75 | 7.86 |
| IPE-LCGA | 45.393611 | -73.938333 | 0 | Uninvaded | 24.26 | 46.26 | 0.431 | 29.76 | 22.8 | 92.0 | 191.9 | 124.80 | 7.90 |
| IB-LCGA | 45.515989 | -73.901314 | 0 | Uninvaded | 9.80 | 42.94 | 0.497 | 6.79 | 20.0 | 89.3 | 80.7 | 52.70 | 7.59 |
| HA-LCGA | 45.614444 | -74.598611 | 0 | Uninvaded | 15.66 | 64.32 | 0.590 | 19.70 | 21.3 | 98.6 | 127.2 | 80.25 | 7.51 |
| GOY-HCGP | 45.329859 | -73.835825 | 4 | 13-16 | 34.74 | 42.39 | 0.393 | 12.82 | 20.3 | 76.8 | 317.3 | 206.05 | 7.73 |
| BEA-HCGP | 45.319491 | -73.852733 | 37 | 13-16 | 37.56 | 66.41 | 0.442 | 19.42 | 19.9 | 94.9 | 307.9 | 199.55 | 8.05 |

**Table S2: Parameters values for the best demographic models**. Population pairs investigated are PB-LCGA with PG-HCGP, IPE-LCGA with BEA-HCGP, and PDC-HCGA with GOY-HCGP. We show the estimated parameters nu1 (scaled effective population size of refuge population), nu2 (scaled effective population size of invaded population), nu1B and nu2B (scaled effective population size after bottleneck in refuge and invaded populations respectively), nu1F and nu2F (scaled effective population size after growth in refuge and invaded populations respectively) m_RI_ (scaled migration rates from invaded to refuge population), m_IR_ (scaled migration rates from refuge to invaded population) and T_S_ (scaled time since the population split), with the upper and lower bounds of the 95% confidence interval shown in brackets.

| **Pair** | **nu1** | **nu2** | **nu1B** | **nu1F** | **nu2B** | **Nu2F** | **m_RI_** | **m_IR_** | **T_S_** |
| --- | --- | --- | --- | --- | --- | --- | --- | --- | --- |
| **PB/**  **PG** | 4.8  [4.6, 5.0] | 14.5  [14.3, 14.7] | 0.01  [-0.03, 0.05] | 0.0004  [-0.0651, -0.0658] | 0.01  [-0.13, 0.13] | 2.7  [-2.3, 7.7] | 0.44  [0.35, 0.45] | 0.01  [-0.03, 0.04] | 14.0  [13.9, 14.1] |
| **IPE/**  **BEA** | 4.1  [2.9, 5.3] | 0.1  [-2.0, 2.2] | - | - | - | - | 0.8  [-1.8, 3.4] | 0.001  [-2.8, 2.8] | 13.6  [9.91, 17.3] |
| **PDC/GOY** | 1.99  [0.03, 3.97] | 3.9  [2.7, 5.1] | - | - | 0.0001  [-2.4870, 2.4872] | - | 1.4  [-1.7,4.5] | 1.4  [-0.4,3.2] | 0.8  [-0.4,2.0] |

**
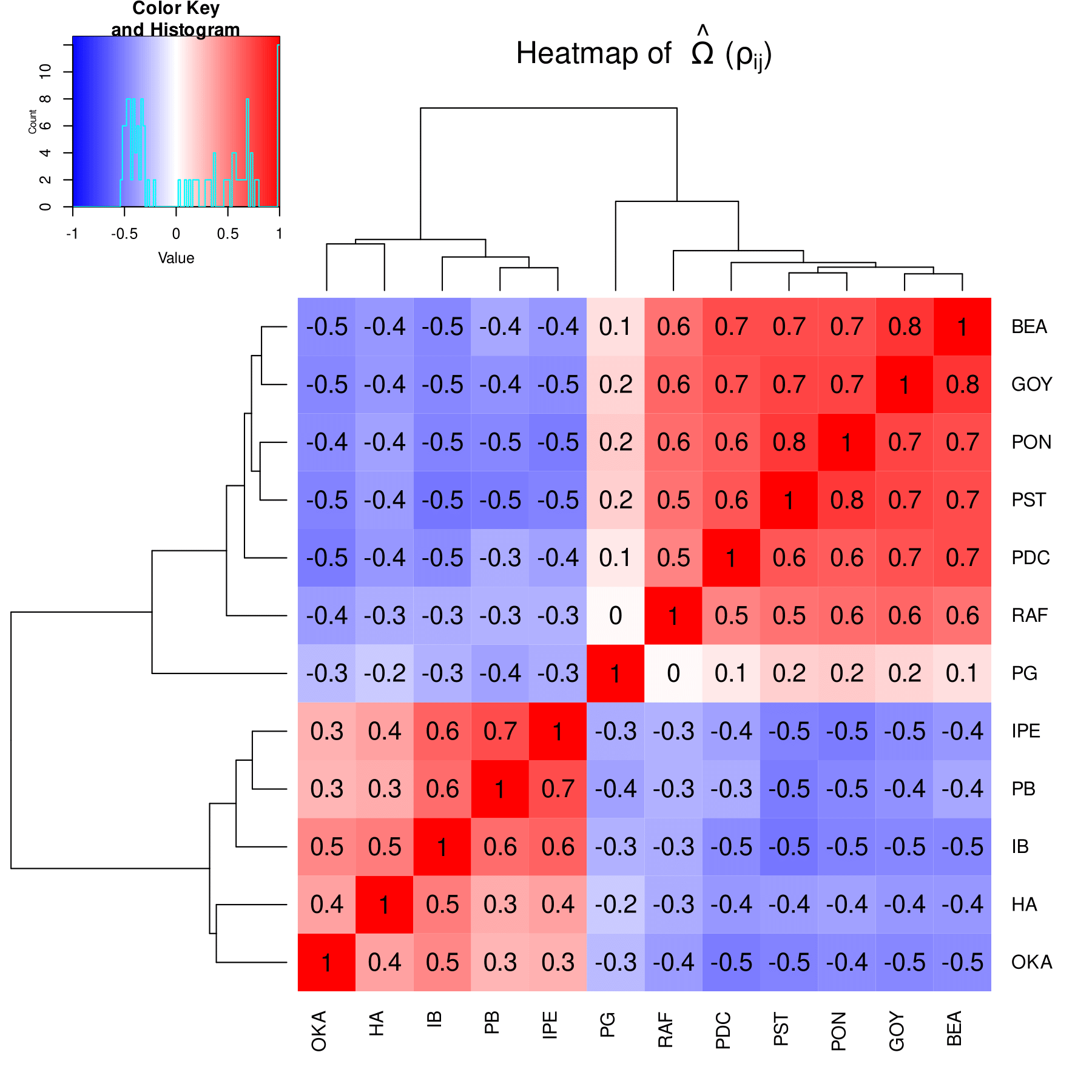
Figure S1:** Heatmap of the scaled covariance matrix Ω (with ρ_ij_ the correlation coefficient between pairs of populations) with hierarchical clustering tree (using the average agglomeration method), obtained from the core model of Baypass.

**Figure S2: Survival rates and fecundity (total number of eggs produced) measured in the reciprocal transplant as a function of COMBO water treatment (low or high calcium concentration), goby cue, and origin water.** Treatments with combo water (High Calcium COMBO HC and Low Calcium COMBO LC) had very low survival and fecundity overall and were thus not included in the statistical analyses presented in the manuscript. Each dot represents a raw data measurement for one population (origins in grey: LCGA, blue: HCGP), summarized by the mean for each treatment (black squares and triangles for treatments with or without round goby cues respectively) and the 95% confidence interval around the mean (bootstrapping method).

**
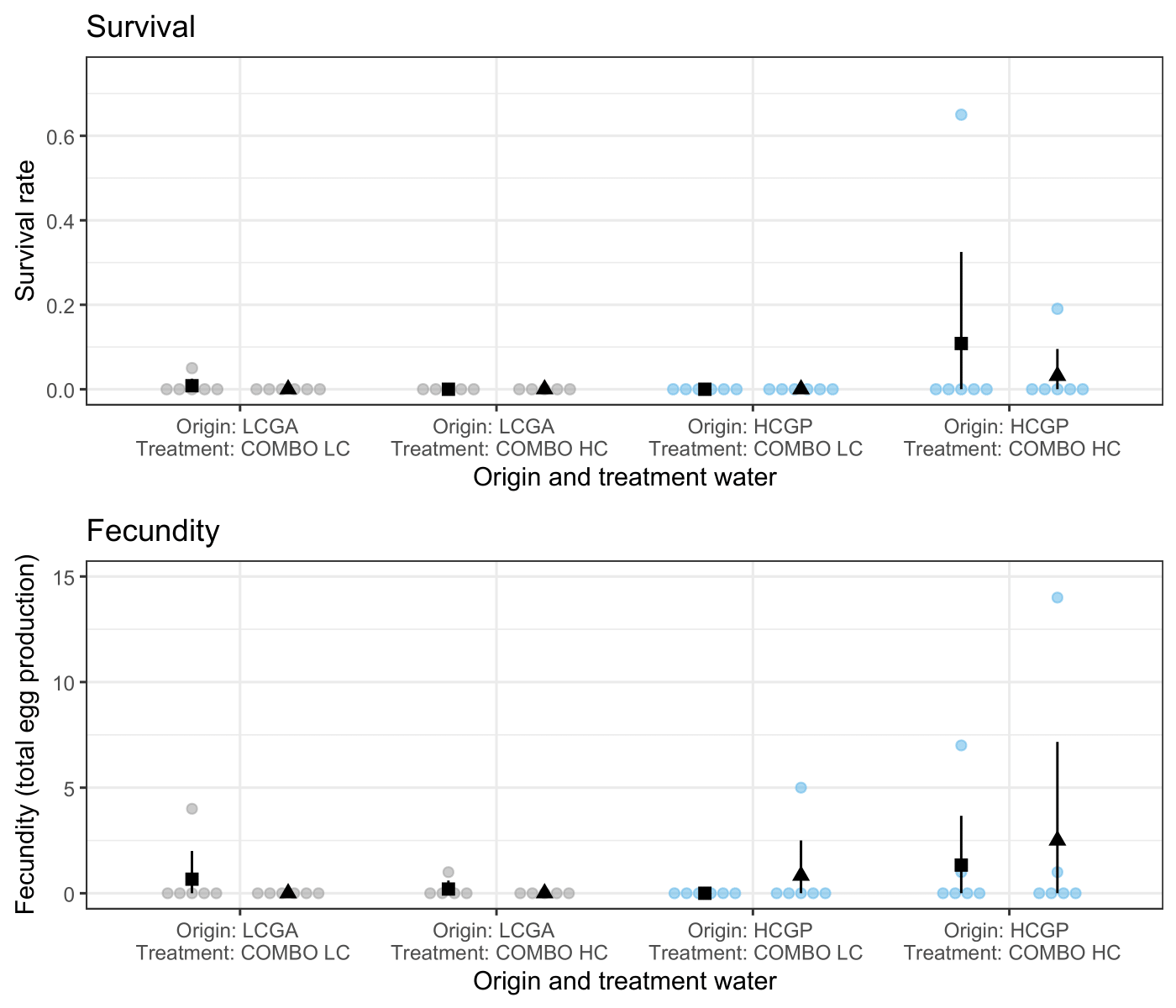
**

**Figure S3: Outliers SNPs identified with the core model in Baypass.** (A) P-values associated with the XtX statistic (on -log10 scale) as a function of the XtX estimate. The horizontal line indicates the threshold of p-values < 0.001. Low XtX values indicate SNPs putatively under balancing selection, and high XtX values indicate SNPs putatively under positive selection (overly differentiated SNPs). (B) Histogram of the p-values derived from the XtX estimate.

**
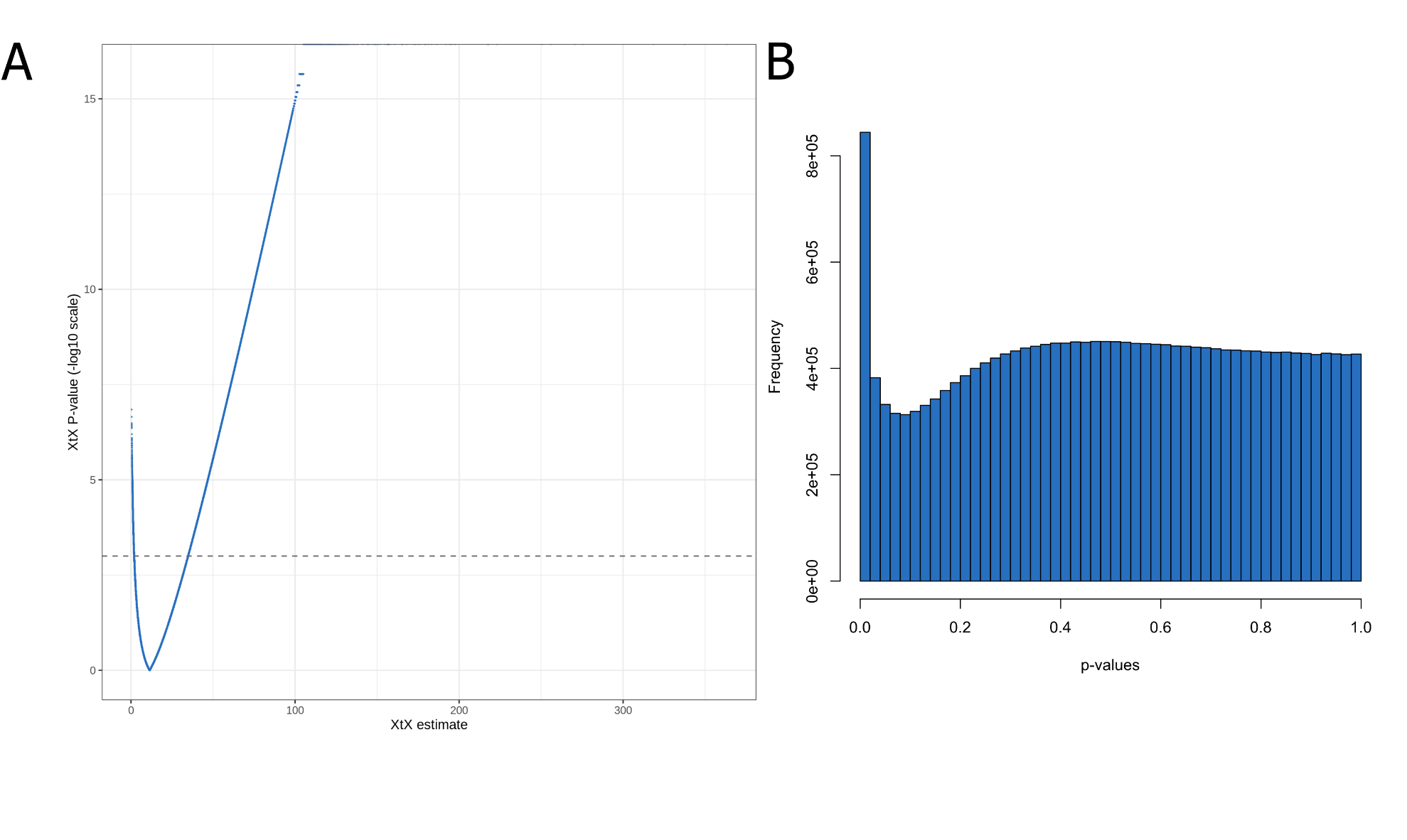
**

**Figure S4: Correlation between selected environmental covariables for the EA analyses with Baypass and RDA.** Inv_status: absence/presence of round gobies, calcium_mg.L: calcium concentration in mg/L

**
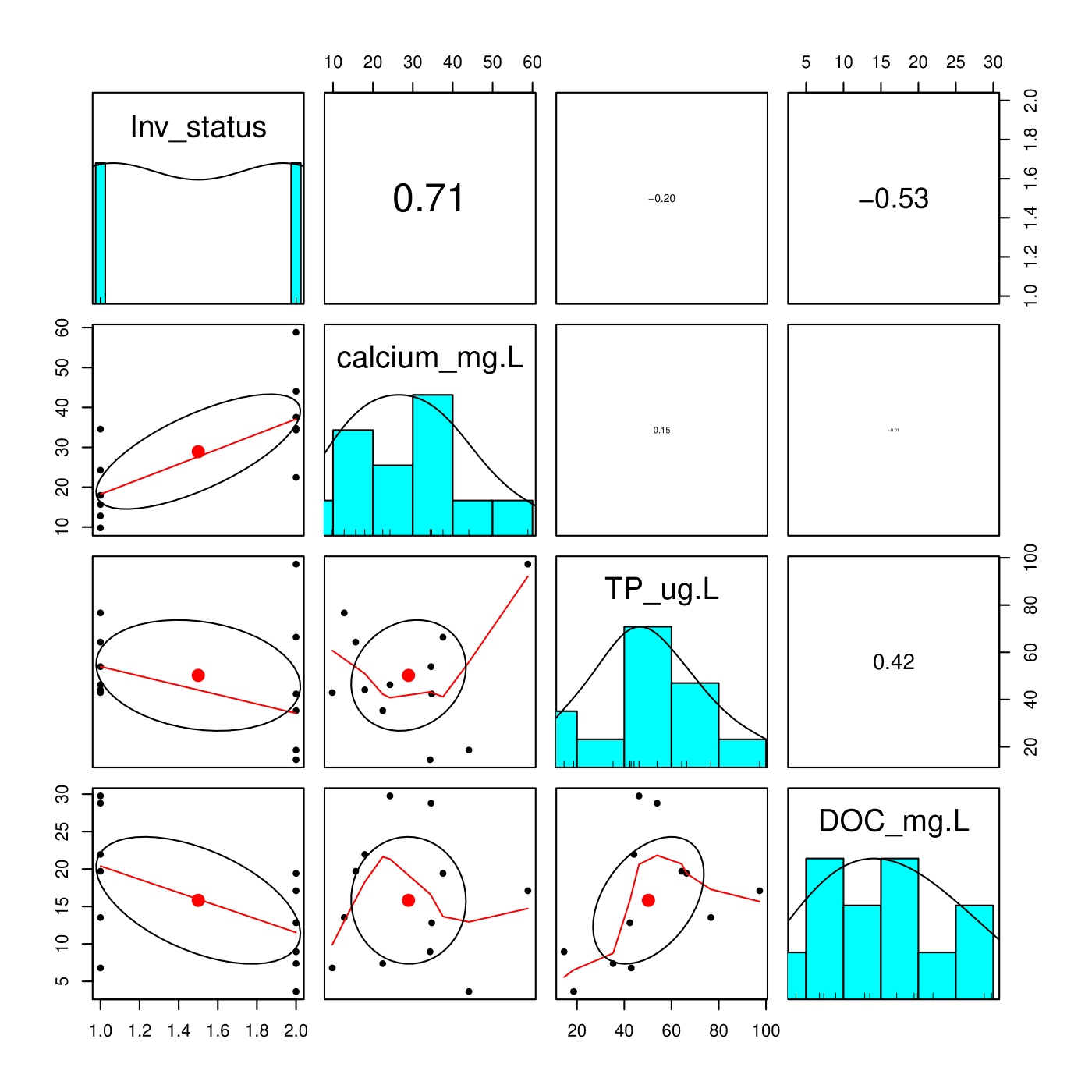
**

**
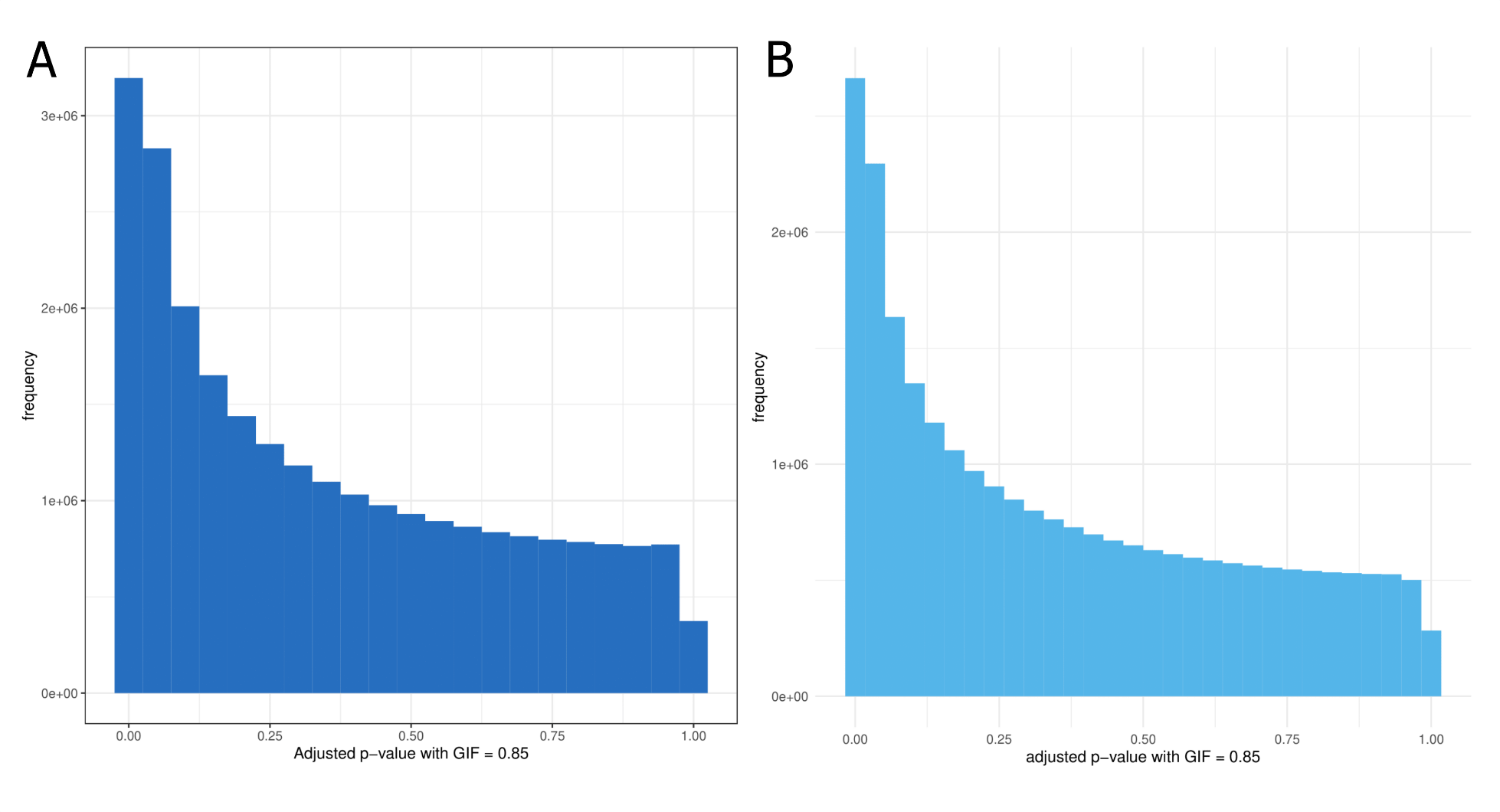
Figure S5: Histograms of the recalibrated p-values output by poolFreqDiff.** P-values were recalibrated based on the empirical-null hypothesis approach, with a genomic inflation factor of λ = 0.85. (A) Recalibrated p-values for the goby presence/absence association. (B) Recalibrated p-values for the calcium concentration association.

**
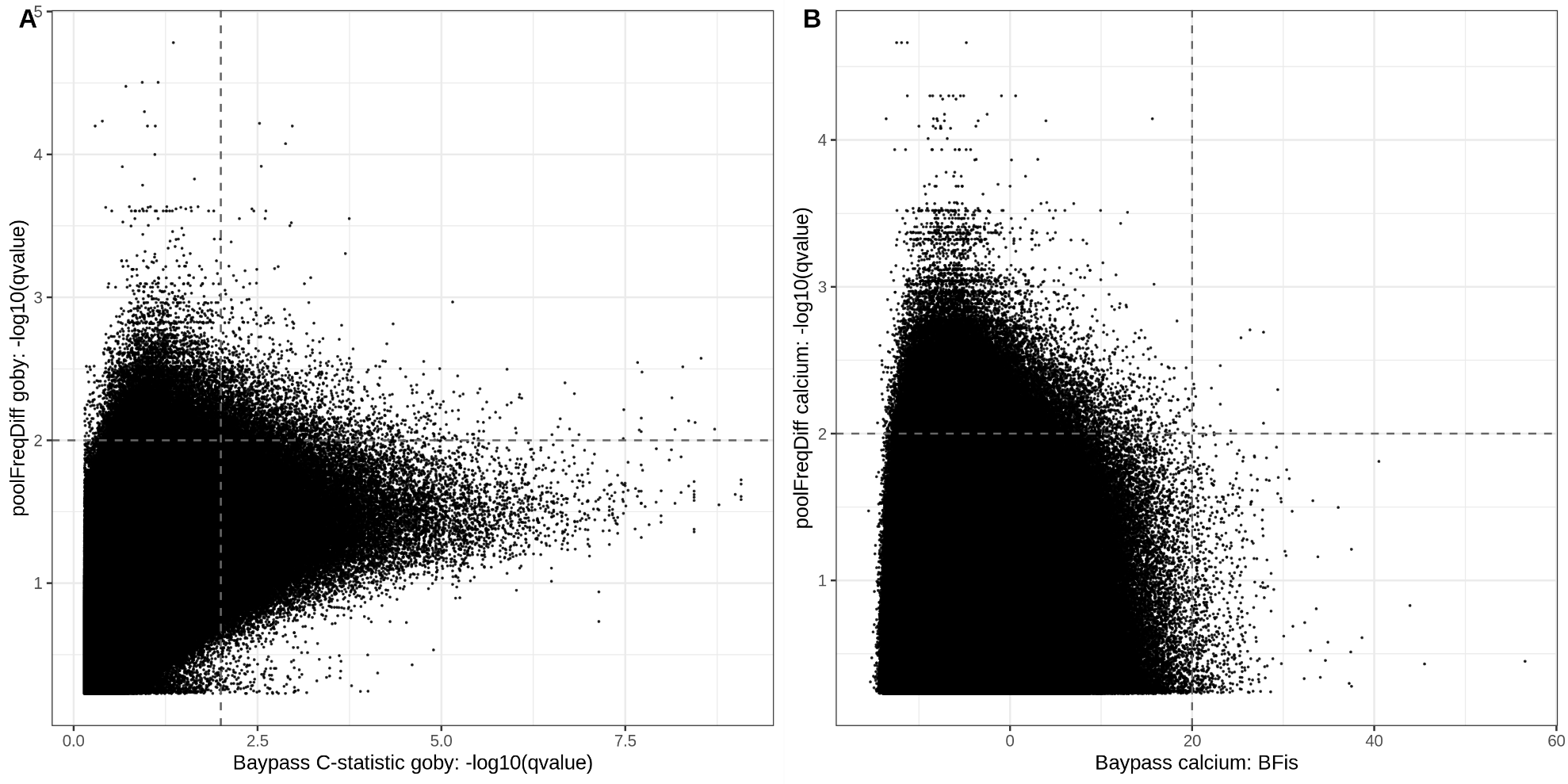
Figure S6:** (A) Biplot of the q-values obtained from the poolFreqDiff analysis testing for consistent differences in allele frequencies between populations from invaded and uninvaded environments, as a function of the q-values derived from the C2-statistic in the STD model in Baypass, assessing the association of SNP allele frequencies with goby presence/absence. The dashed vertical and horizontal grey lines indicate the FDR of α 0.01. (B) Biplot of the q-values for the poolFreqDiff analysis comparing populations of low and high calcium habitats as a function of the Bayes Factor (BFis) from the Baypass STD model (median of three independent runs) testing the association with calcium concentration. The dashed vertical line (BFis > 20 dB) indicates outlier SNPs significantly associated with the calcium covariable and the horizontal line gives the q-value FDR of α 0.01.

**Figure S7: Demographic models tested with dadi.** Visual model of the most complex demographic model tested with a population split, then bottlenecks in both populations, followed by exponential recovery, with migration being kept constant throughout (A). Parameters nu1 and nu2 are the scaled effective population sizes after the split for the refuge and invaded populations respectively, nu1B/nu1F and nu2B/nu2F represent the scaled effective population sizes of the refuge and invaded populations after the bottlenecks and the recovery respectively. T_S_ is the scaled time between the split and the bottleneck, and T_B_ is between the bottleneck and the present. m_RI_ is the scaled migration rate from the invaded population toward the refuge population, and inversely for m_IR_. Simpler models investigated include (B) bottleneck and growth only in the invaded population (constant N_e_ for the refuge population) with uneven migration, (C) only bottlenecks in both populations without recovery, with uneven migration, (D) bottleneck only in the invaded population (constant N_e_ for the refuge population) with uneven migration, (E) a simple population split at T_S_ with uneven migration, (F) a population split at T_S_ with symmetric migration and (G) a population split at T_S_ without migration.

**
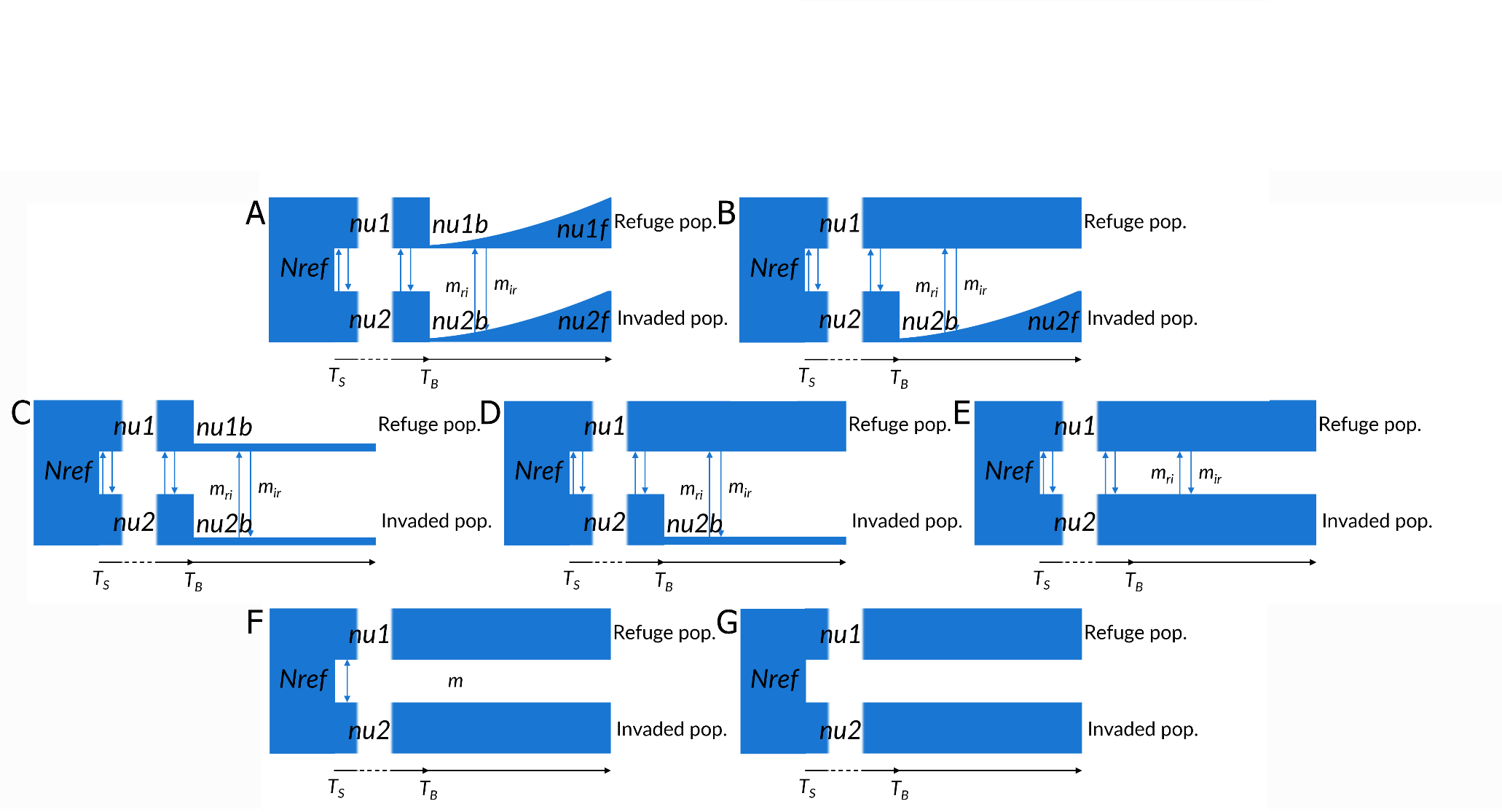
**

**Figure S8: Random effect of the populations of origin from the GLMM model for survival (binomial distribution and logit link function).** Random effect deviations showing shifts of populations relative to the fixed intercept (0). The circles’ color indicates the habitat or origin: the Ottawa River (grey, LCGA) and the St. Lawrence River (blue, HCGP), except for PDC (HCGA, wetland refuge) and RAF (LCGP)


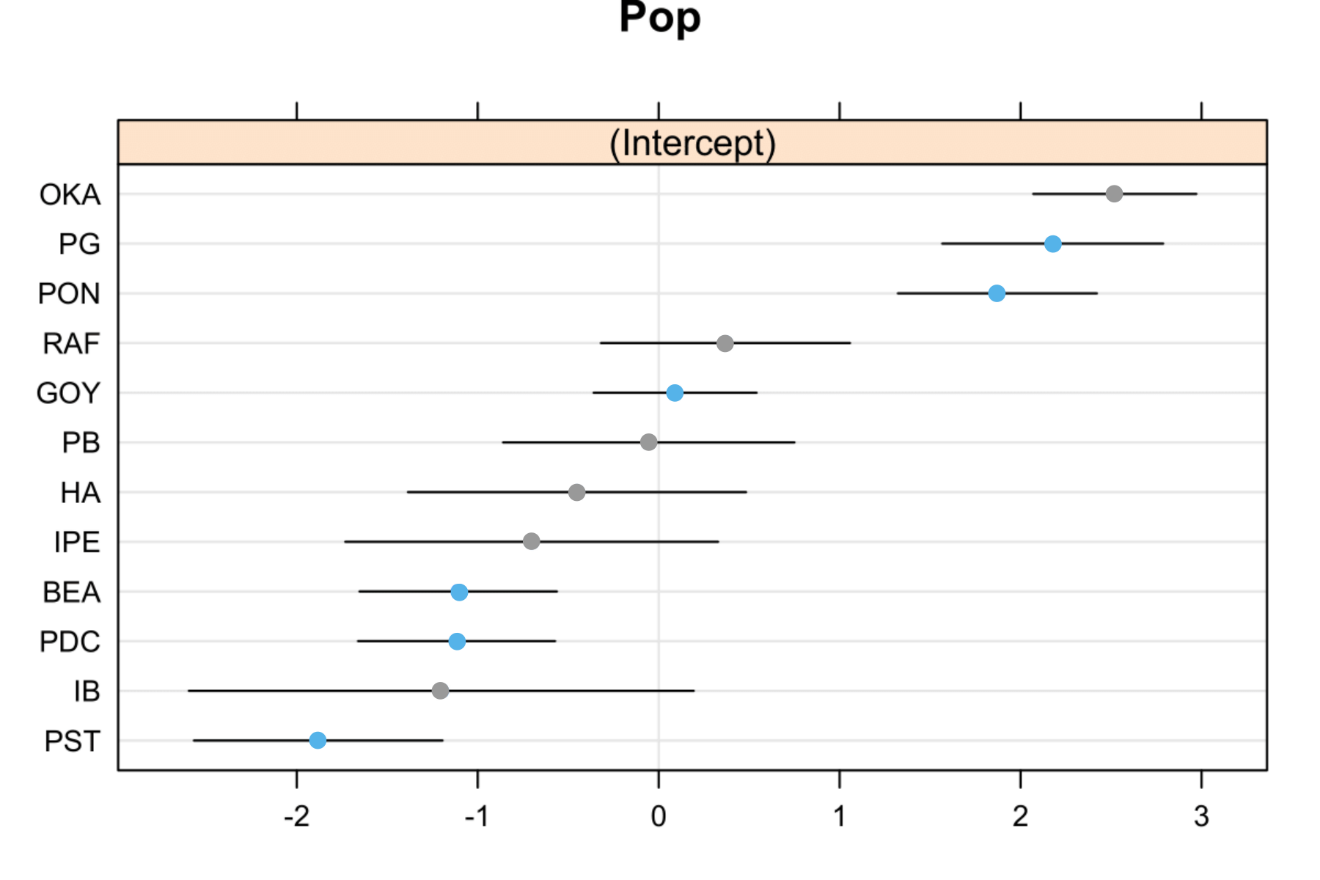


**Figure S9: Venn diagram of the outliers from the Environmental association analyses.** Compared methods are the Baypass STD model (Calcium concentration) for the three replicate runs, the Baypass C-statistic from the STD model (Goby presence), and the poolFreqDiff analyses of consistent allele frequency differences between environment types, for both the calcium concentration and invasion status (presence/absence). Outlier SNPs in common between the analyses are shown in the shaded areas.

**
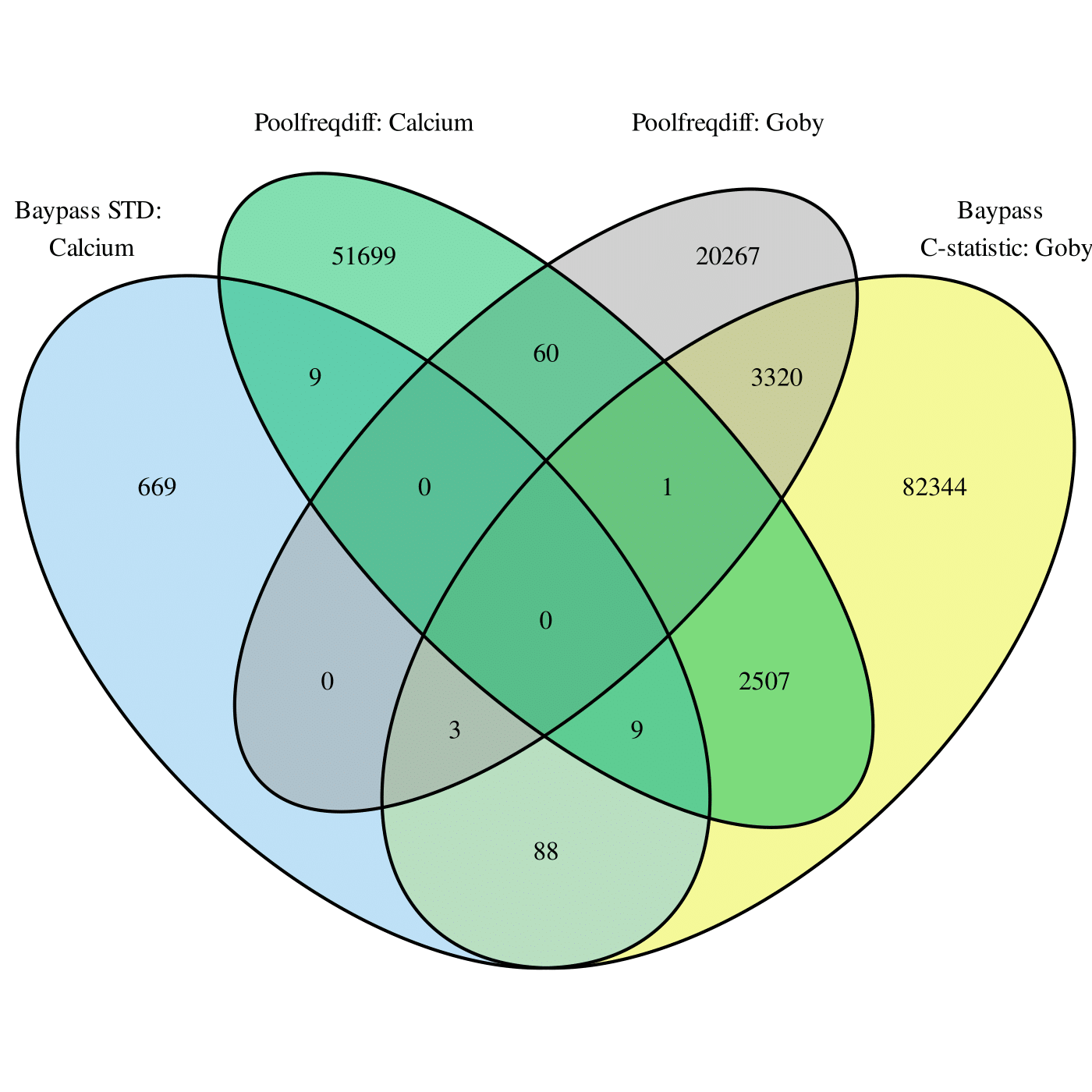
**

**Figure S10: Venn diagram of the outlier and Environmental association analyses.** Outliers SNPs from Baypass (Core and STD models, C-statistic for the association with goby absence/presence) and the poolFreqDiff analysis of consistent allele frequency differences between environment types. Outlier SNPs in common between the three methods are shown in the shaded areas.

**
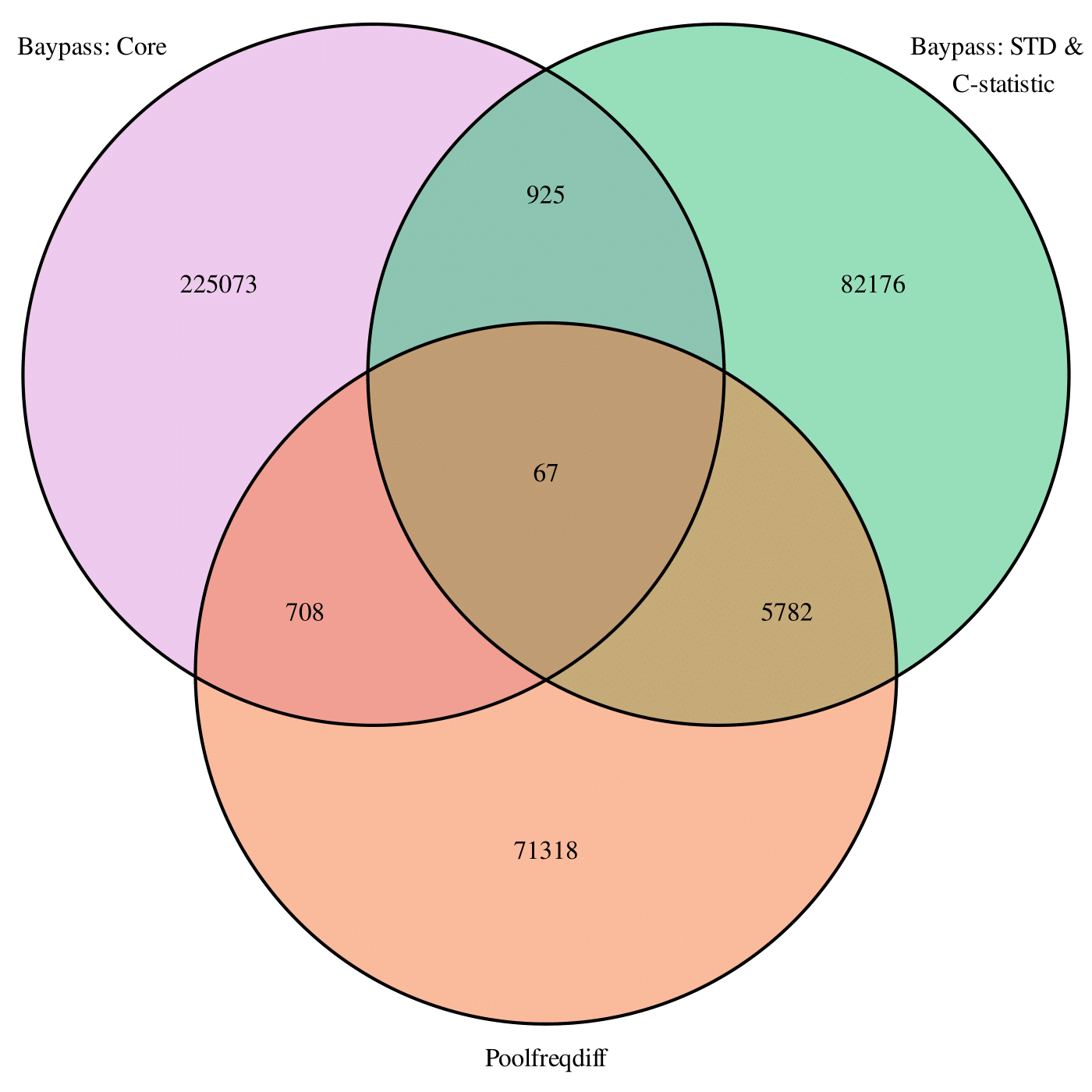
**

**
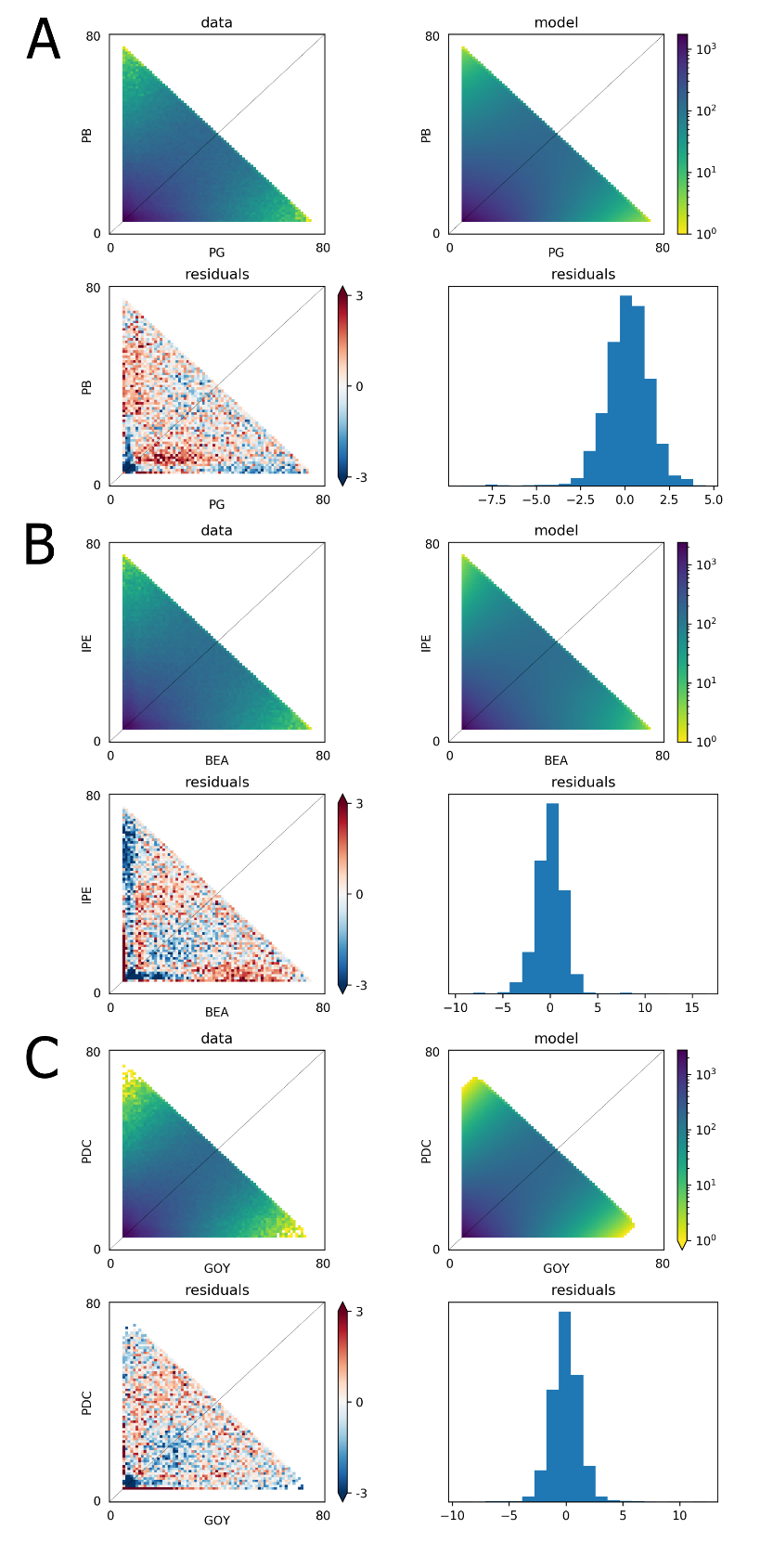
Figure S11: Best demographic models tested with dadi.** (A) Results for the model with bottleneck followed by growth in PG-HCGP and PB-LCGA. Top: Folded joint site frequency spectrums (SFS) for a haploid sample size of 80 for the observed data (left) and the model (right). The colored scale indicates the logarithm of the number of sites for a given read count. Note that the data was masked from 0 to 5. Bottom: residuals of the normalized differences between the observed data and the model (left), shown as a histogram (right). (B) Results for the model of split with uneven migration in BEA-HCGP and IPE-LCGA. (C) Results for the model of bottleneck followed by growth only in the invaded population GOY-HCGP (constant *N_e_* for PDC-HCGA).

**Figure S12: Pattern of isolation by distance and by the environment between the study populations. The genetic distance** F_ST_/(1-F_ST_) is based on the pairwise F_ST_ matrix from putatively neutral SNPs (outliers excluded). (A) Significant positive correlation (Mantel test: p = ${8\times10}^{-4}$, r^2^ = 0.285) between the log of the geographical distance (in m, 2D distribution of populations) and the linearized genetic distance F_ST_/(1-F_ST_). (B) Significant positive correlation (Mantel test: p = 0.044, r^2^ = 0.078) between the Mahalanobis environmental distance and the linearized genetic distance F_ST_/(1-F_ST_).

**
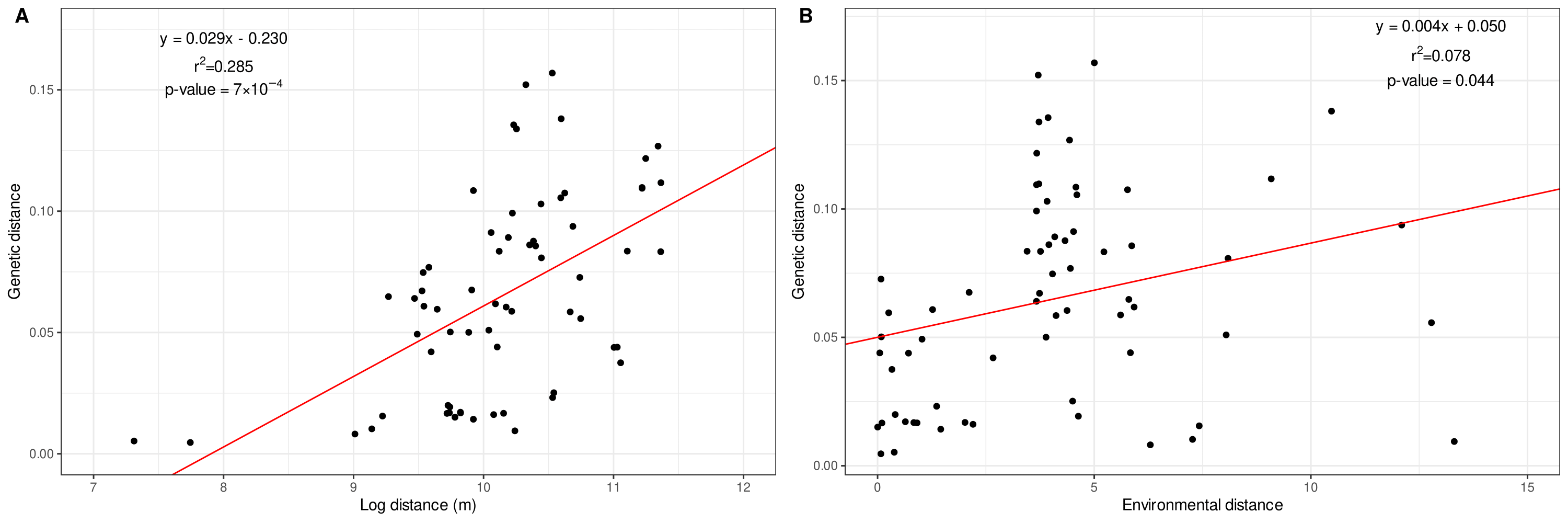
**
